# Integrated Multi-Tissue Transcriptomics Reveals Antagonistic Pleiotropy in Aging and Alzheimer’s Disease

**DOI:** 10.34133/csbj.0134

**Published:** 2026-06-08

**Authors:** Rana Salihoglu, Şehnaz Can, Thomas Dandekar, Elena Bencurova

**Affiliations:** ^1^ Department of Bioinformatics, Julius-Maximilians-Universität Würzburg, Würzburg, Germany.; ^2^ Department of Bioengineering, Marmara University, Istanbul, Turkey.

## Abstract

Aging is the strongest risk factor for Alzheimer’s disease (AD); however, some individuals age without major cognitive decline, suggesting that resilience and vulnerability may be associated with distinct molecular trajectories. To investigate these trajectories, we performed an integrated transcriptomic analysis of human dermal fibroblasts (GSE113957) and multi-region brain profiles (GSE48350), extending previous dataset-specific studies that focused primarily on age prediction, regional variation, or synaptic/immune signatures. Healthy aging and AD were compared within a novel antagonistic pleiotropy (AP) framework. This approach prioritized genes and candidate transcriptional regulators with opposing age and disease-associated expression patterns. Across tissues, healthy aging was associated with relative preservation of metabolic, mitochondrial, and lipid-homeostatic programs, whereas AD was associated with suppression of these programs alongside greater inflammatory and immune pathway activity. AP-Vulnerability genes (Age↓/AD↑), including TAC1, FREM3, and SLC25A46, declined with age but were induced in AD. Conversely, AP-Resilience genes (Age↑/AD↓), including PTH2, PPDPF, and NEFH, increased during healthy aging but were reduced in AD. Pathway analyses suggested an association between metabolic programs and resilience, and between immune activation and vulnerability. Transcription-factor inference prioritized PPARG, NFE2L2, and TEAD4 as candidate resilience-associated regulators, showing directionally opposite patterns relative to immune- and developmental-related regulators in AD.

## Graphical Abstract

**Figure:**
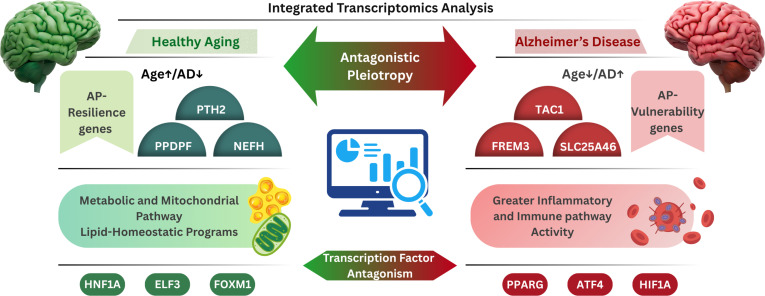

## Introduction

Aging is the predominant risk factor for Alzheimer’s disease (AD), but the molecular programs that separate cognitive resilience from neurodegeneration remain incompletely defined. Epidemiological and mechanistic studies show substantial overlap between AD risk factors and major features of late-life biology, including vascular dysfunction, physical inactivity, hearing loss, depression, and social isolation [1,2]. Network-based analyses of metabolic and neuropsychiatric comorbidity further support this idea. Systemic risk states and brain-associated disorders converge on shared pathway architecture, including insulin, inflammatory, calcium, mitogen-activated protein kinase, estrogen, apoptosis-related, and connected signaling pathways [3]. These observations support a model in which AD is a distinct pathological trajectory different from aging, and only a subset of older individuals progress from age-associated molecular changes to a trajectory toward clinical neurodegeneration.

Antagonistic pleiotropy (AP) provides a useful conceptual framework for interpreting these divergent aging trajectories. While classical AP is defined by opposing effects on fitness across the lifespan, here AP is operationally characterized by genes with directionally opposing expression trajectories between healthy aging and AD, which may represent the molecular correlations of such pleiotropic programs. Recent computational work on pleiotropic expression quantitative trait loci has shown that variants affecting both traits and gene expression are enriched in enhancers and transcription-factor (TF) binding regions, supporting a regulatory basis for pleiotropic transcriptomic effects [4]. Cross-disorder genetic and microbiome analyses also indicate that pleiotropic gene sets can connect brain-related and systemic phenotypes through immune regulation and synaptic signaling, reinforcing the relevance of pleiotropy to complex late-life disease biology [5]. In this model, biological programs that are adaptive earlier in life (e.g., growth, repair, and stress responsiveness) may become maladaptive later (e.g., chronic inflammation, synaptic dysfunction, or metabolic decline) [6]. Identifying such programs in humans requires integrative, cross-tissue transcriptomic comparisons spanning healthy aging and neurodegenerative disease states [7,8]. Computational genetics approaches that jointly model multiple brain imaging traits from GWAS (genome-wide association study) summary statistics illustrate the broader value of multivariate and summary statistics-based integration for resolving brain-relevant genetic architecture [9].

In the brain, healthy aging is commonly associated with adaptive regulation of oxidative stress responses, metabolic homeostasis, and maintenance of neuronal/synaptic function [10–13]. In contrast, AD is marked by amplified neuroinflammatory signaling, mitochondrial dysfunction, and progressive synaptic loss [14–16]. Whole-genome pathway analyses of AD similarly prioritize biological themes that include immunity and inflammation, metabolism, protein homeostasis, DNA/RNA and epigenetic regulation, synapse/structure, and cell-cycle pathways [17]. The coexistence of convergent and divergent processes raises a central hypothesis: Pathways that support resilience in normative aging may be selectively attenuated or directionally inverted in AD, consistent with AP [18,19].

This framework motivates parallel, tissue-aware comparisons of healthy aging and AD to identify genes and regulatory systems whose age-associated trajectories are weakened or reversed in disease [20–23]. Here, we integrate transcriptomic data from human fibroblasts (GSE113957) and 4 cortical regions (entorhinal cortex, hippocampus, post-central gyrus, and superior frontal gyrus; GSE48350) to define shared versus divergent expression programs across physiological and pathological aging. The fibroblast dataset (GSE113957) was originally published for chronological age prediction using machine learning, rather than for studying AD or cross-tissue antagonism [24], and is used here as a peripheral aging context instead of a direct proxy for brain aging. In parallel, the 2 Berchtold studies motivating our brain context reported that normal brain aging is strongly region- and sex-dependent at the transcriptome level, and that synaptic genes are extensively down-regulated across multiple regions in both aging and AD, in line with prominent neuronal/synaptic program loss in AD [25,26]. Using differential expression, meta-analysis, and weighted gene coexpression network analysis (WGCNA), we first classified AP-like transcriptomic candidates into 2 operational directional classes: AP-Resilience (Age↑/AD↓) and AP-Vulnerability (Age↓/AD↑). We next identified candidate regulatory hubs, including genes, potentially linked to metabolic, stress-response, immune, and synaptic programs.

Collectively, these analyses indicate that selected transcriptomic programs diverge between healthy-aging and AD-associated contrasts. Although no direct evidence of failed compensation, maladaptive induction, or causal loss of homeostatic programs, by separating shared aging–AD expression changes from opposing patterns, the AP-centered framework provides a transcriptomic strategy for distinguishing AD-associated aging signals from regulatory programs more closely aligned with non-AD aging. This computational network analysis, supported by independent transcriptomic validation, nominates candidate genes, pathways, and upstream regulators that may help prioritize future mechanistic studies of aging-associated resilience and AD vulnerability.

## Methods

### Study design, datasets, and cohort definition

Transcriptomic datasets were obtained from the Gene Expression Omnibus (GEO) and downloaded using the Cat-E tool [27]. Dermal fibroblast aging was analyzed using the GSE113957 dataset, which provides raw RNA-sequencing (RNA-seq) counts and phenotype annotations [24]. AD and healthy brain aging were analyzed using the GSE48350 dataset, generated on the Affymetrix Human Genome U133 Plus 2.0 platform (GPL570). SeriesMatrix files were imported, and phenotype fields were parsed to extract age, sex when available, brain region, and disease status. Brain regions were harmonized as entorhinal cortex, hippocampus, postcentral gyrus, and superior frontal gyrus.

**Fig. 1.**
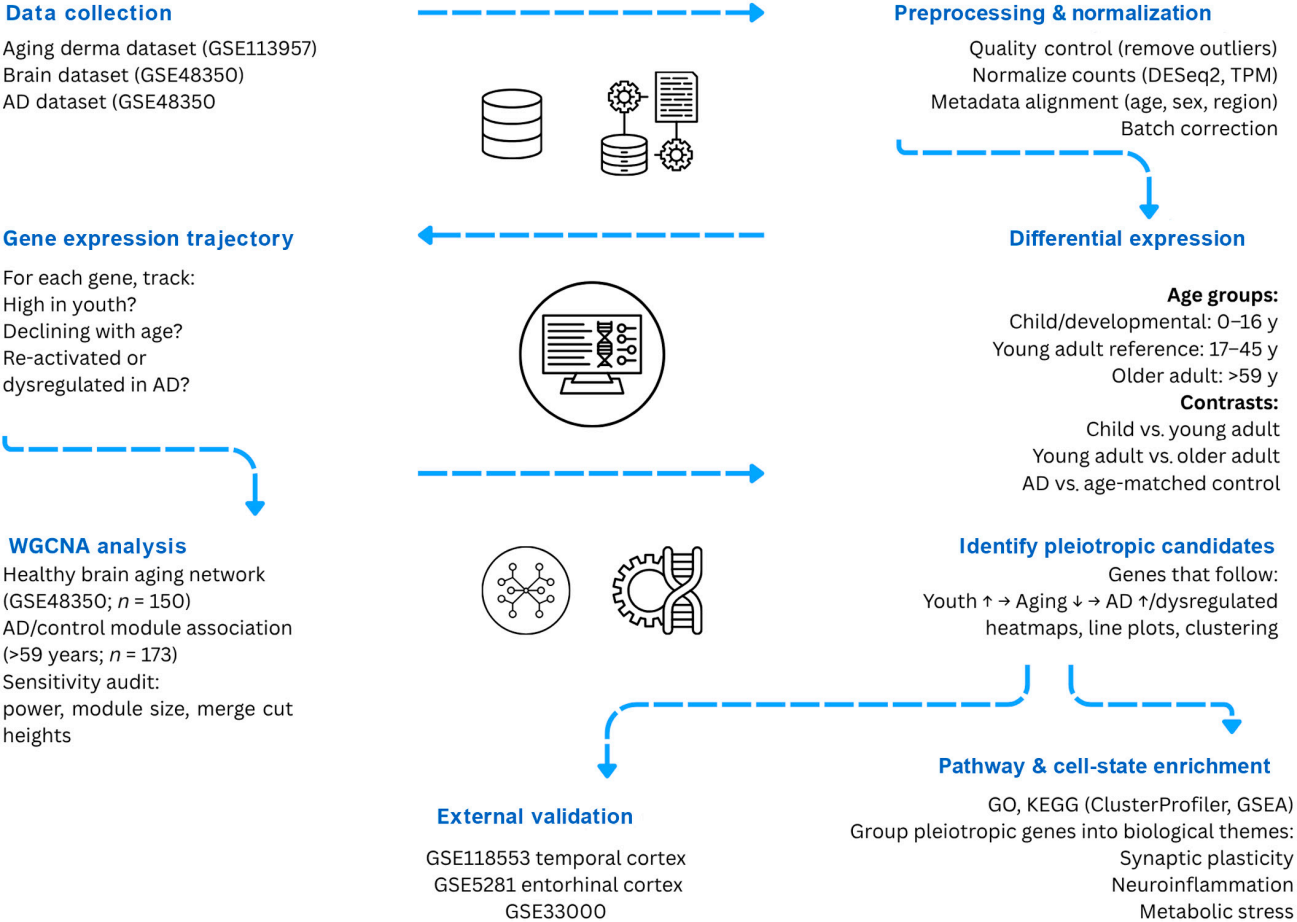
Overview of the analytical workflow for identifying AP-like transcriptomic patterns in aging and AD.

Age groups were defined a priori as child/developmental (0 to 16 years), young adult/adult reference (17 to 45 years), midlife (46 to 59 years), and older adult (>59 years). These thresholds were used as operational analysis boundaries for comparing predefined age strata across tissues. Midlife samples were excluded from the primary adult-aging contrast to reduce mixing of transitional midlife biology with young-adult or older-adult profiles. Because dermal and neural tissues have distinct developmental and aging trajectories [28], GSE113957 was interpreted as a peripheral developmental/aging context, not as a direct proxy for brain aging (Fig. 1).

For GSE113957, developmental analyses compared children (0 to 16 years) with young adults (17 to 45 years), whereas adult fibroblast aging analyses compared young adults (17 to 45 years) with older adults (>59 years). The sex-adjusted adult-aging audit retained 98 samples: 42 young adults and 56 older adults, including 28 female and 70 male samples. For GSE48350, AD contrasts were restricted to individuals aged >59 years and analyzed region-wise. The brain analysis cohort comprised 173 samples, including 93 controls and 80 AD cases, distributed across entorhinal cortex (*n* = 33), hippocampus (*n* = 44), postcentral gyrus (*n* = 49), and superior frontal gyrus (*n* = 47) (see also Table S1).

Independent external validation of prioritized AP candidates was performed using 3 additional AD transcriptomic cohorts: GSE118553 temporal cortex, GSE33000, and GSE5281 entorhinal cortex. These datasets were not used for AP candidate discovery or prioritization. Instead, they were analyzed separately to assess whether prioritized AP genes showed AD-associated expression changes in the expected direction. External validation was therefore restricted to expression-level reproducibility of AP-class directionality; there was no genotype-based or neuropathology-adjusted replication.

Cohort definitions, sample counts, age ranges, sex distributions, and available covariates are summarized in Table S1. Available covariates included age, sex, brain region, and disease status, where provided and estimable. APOE genotype, Braak/CERAD (Consortium to Establish a Registry for Alzheimer’s Disease) scores, medication, comorbidity, ethnicity/geographic background, and detailed cognitive metadata were not uniformly available across the discovery and validation datasets and were not imputed. Established AD-risk genes such as APOE, ABCA7, BIN1, SORL1, TREM2, CLU, PICALM, and CR1 were inspected only as expression-level contextual markers when measurable and were not forced into AP classes.

### Computational environment and reproducibility

All analyses were performed in R (v4.2.2) using Bioconductor packages including Biobase [29] (v2.58.0), GEOquery [30] (v2.66.0), limma [31] (v3.54.0), and DESeq2 [32]. Package installation and version control were managed with BiocManager. Gene identifier mapping used AnnotationDbi [33] and org.Hs.eg.db. Data wrangling used dplyr, tidyr, tibble, and stringr; visualizations were generated with ggplot2, ggrepel, plotly, heatmaply, htmlwidgets, VennDiagram, and grid. Functional analyses used clusterProfiler [34], GSEABase, and GSVA. Random seeds were fixed for stochastic procedures [e.g., Uniform Manifold Approximation and Projection (UMAP)]. Intermediate tables, model objects, and figures were written programmatically to a single results directory to preserve a complete computational record.

### Preprocessing and differential expression analyses

For GSE113957 RNA-seq data, raw count matrices were filtered to remove lowly expressed genes before differential expression analysis. Adult fibroblast aging was assessed among samples annotated as normal by comparing young adults aged 17 to 45 years with older adults aged >59 years. Differential expression was modeled with DESeq2 [32] using age group as the primary factor, followed by standard normalization, dispersion estimation, and Wald testing. Sex metadata were available and were reported for cohort transparency; however, sex was not included in the primary fibroblast model because the adult-aging subset was sex-imbalanced, and sex-stratified modeling substantially reduced effective sample size. Accordingly, the fibroblast analysis was treated as a peripheral age-associated contrast, with sex-specific aging inference reserved for balanced cohorts.

Gene identifiers were harmonized to HGNC (HUGO Gene Nomenclature Committee) symbols before downstream integration. Unless otherwise specified, statistical significance was defined as false discovery rate (FDR) < 0.05, with FDR < 0.01 used to highlight top-ranked genes. Variance-stabilized expression values were used for exploratory quality-control visualizations, including principal components and low-dimensional embedding analyses. Additional diagnostic outputs included differential expression summary plots and heatmaps of top-ranked genes. For GSE48350 microarray data, expression matrices were imported from GEO, log-transformed when required, normalized, and summarized at the gene level after probe-to-symbol annotation. Differential expression was estimated with limma [31] using empirical Bayes moderation.

AD-associated expression changes were modeled separately within each brain region using disease status as the primary variable, with analyses restricted to regions containing sufficient control and AD samples. Brain aging contrasts were performed among control samples only and were stratified by brain region, comparing older adults with young-adult reference samples. FDR < 0.05 was used as the primary significance threshold.

To reduce the influence of technical artifacts and outlying samples, microarray subsets underwent multistep quality control before final modeling. This included principal components analysis (PCA)-based outlier assessment, sample-connectivity checks, expression-distribution diagnostics, near-duplicate screening, and sex-marker consistency checks using X-inactive specific transcript (XIST) and Y-linked genes. Samples failing predefined quality-control criteria were excluded before final normalization and differential expression analysis. This workflow was designed to preserve dataset-specific modeling while avoiding direct merging of RNA-seq and microarray expression values.

### Cross-condition integration and meta-analysis

Differential expression outputs from fibroblast aging, brain aging, and AD contrasts were harmonized by gene symbol, log₂FC, and adjusted *P* value using readr-based import pipelines. Cross-platform integration was performed only after dataset-specific normalization and differential expression modeling, using signed effect sizes, *P* values, and meta-analytic statistics. This design reduces the risk that platform-specific dynamic range, gene detectability, or noise structure introduces artificial directionality into the integrated AP signal. Where needed, *P* values were re-adjusted by Benjamini–Hochberg correction; Ensembl identifiers were cleaned and mapped to HGNC symbols. Genes were classified as up- or down-regulated using the direction of log₂FC and statistical significance (default FDR < 0.05; relaxed to FDR < 0.10 in low-signal settings).

Overlap strength between contrasts was quantified by intersection size, Jaccard index, and Fisher’s exact tests with Benjamini–Hochberg correction. Effect-size concordance was evaluated by Spearman correlations of log₂FC across tissues and summarized as heatmaps.

To derive condition-level consensus signals, per-contrast *P* values were converted to *Z* scores and combined by Stouffer’s method; combined *Z* scores were converted to 2-sided meta *P* values and FDR-adjusted. For AD, region-specific contrasts were incorporated with predefined weights where applicable. Consensus effect size was defined as the sign-aligned mean log₂FC across contributing contrasts. For each gene and condition, we retained consensus log₂FC, meta-analytic FDR, and the number of contributing contrasts. Consensus up/down sets were defined by meta-analytic significance and the sign of consensus log₂FC.

### Gene classification, prioritization, and AP metrics

Condition-specific consensus tables were joined by gene symbols to assign genes to 7 mutually exclusive classes: Shared Up, Shared Down, discordant Age↑/AD↓, discordant Age↓/AD↑, AD-specific, Age-specific, and nonsignificant. Prioritization used composite metrics. For shared classes, a shared score was defined as the minimum absolute log₂FC across conditions multiplied by the product of −log_10_(FDR) values and by the minimum number of contributing contrasts. For discordant and condition-specific classes, a divergence score was defined as the absolute log₂FC difference multiplied by −log_10_(FDR) and scaled by the number of contributing contrasts. A unified priority value uses a shared score for shared classes and a divergence score otherwise.

Aging pleiotropy was quantified as the fraction of aging contrasts with log₂FC sign matching the aging consensus sign (and in a restricted variant, only significant contrasts). In AD, opposition to aging was assessed per gene by counting regional contrasts with opposite sign relative to aging consensus and applying a one-sided binomial test.

Discordant classes were further labeled as AP-Resilience (Age↑/AD↓) and AP-Vulnerability (Age↓/AD↑). Candidate sets were first defined using primary FDR/effect-size criteria; if either set was empty, thresholds were relaxed by a predefined sequence until both sets were populated. Genome-wide ranked vectors for downstream enrichment were derived from consensus meta-analysis using signed significance statistics that preserved effect direction and magnitude.

### Covariate and brain-only sensitivity audits

To address potential covariate imbalance and dependence on peripheral aging information, we performed add-on sensitivity analyses without replacing the primary differential expression or AP-prioritization outputs. First, the adult fibroblast aging contrast was re-estimated in GSE113957 using a sex-adjusted DESeq2 model restricted to the predefined adult-aging comparison of young adults aged 17 to 45 years versus older adults aged >59 years; children aged 0 to 16 years and midlife samples aged 46 to 59 years were reported for transparency but excluded from this adult-aging covariate audit. Sex was included only when available and when the design matrix was estimable. Second, the AD brain contrast was re-estimated using limma/empirical Bayes models with disease status as the primary term and available covariates, including sex, age, and brain region, included only when the design matrix remained full-rank. Third, to test whether AP classification depended on dermal fibroblast aging information, we performed a brain-only dermal-exclusion sensitivity audit. Region-wise healthy brain aging differential expression results from entorhinal cortex, hippocampus, postcentral gyrus, and superior frontal gyrus were combined into a signed brain-aging meta-effect table, and the corresponding AD versus age-filtered control contrasts were combined into an AD-brain meta-effect table. Brain-only AP-Resilience was assigned to genes with positive brain-aging and negative AD-brain meta-effects, whereas brain-only AP-Vulnerability was assigned to genes with negative brain-aging and positive AD-brain meta-effects. Original AP candidates were then compared with their brain-only classifications to quantify testability, same-class retention, class switching, and loss of AP-like directionality after excluding dermal aging information. These audits were used to evaluate the robustness of effect direction and AP classification, not to establish causal AP.

### Pathway and TF analyses

Pathway-level enrichment used fgsea on ranked consensus statistics for aging and AD. Gene sets were obtained from MsigDB via msigdb [35], focusing on Hallmark and Gene Ontology (GO) Biological Process collections. Gene annotations were mapped to current HGNC symbols and converted to pathway-to-gene lists. fgseaMultilevel was used to estimate normalized enrichment scores (NESs) and Benjamini–Hochberg adjusted *P* values, with gene-set size bounds of 15 to 2,000; when convergence failed or missing values occurred, fgseaSimple with increased permutations was used. To evaluate concordance versus antagonism between aging and AD, NES tables were merged by pathway and classified by NES sign combinations (same-direction versus antagonistic quadrants).

TF activity was inferred from consensus signatures. Where consensus outputs were unavailable, raw differential tables were re-aggregated with Stouffer *Z* meta-analysis using predefined AD regional weights. Optionally, a curated AP gene list was used to restrict or weight inference. Per condition, signatures were constructed from signed −log_10_(*P*) values using consensus log₂FC direction in 3 modes: genome-wide, AP-only, and AP-weighted. Regulatory interactions were obtained from DoRothEA (confidence A-C, signed weights), filtered to genes present in the signature universe, and summarized by quality control (QC) metrics (targets per TF, overlap proportions, AP overlap). TF activities were estimated using decoupleR (WSViper); if unavailable or nonconvergent, a fallback *z*-scored weighted target-mean approach was applied. Enrichment of AP regulation among highly active TFs was tested by one-sided Fisher’s exact tests, followed by Benjamini–Hochberg correction, and regulons with FDR < 0.1 were flagged.

### WGCNA analysis and AP module/gene scoring

Weighted gene coexpression network analysis (WGCNA; v1.72-1) [36] was applied to preprocessed GSE48350 brain transcriptomes to evaluate whether aging- and AD-associated signals were organized within reproducible coexpression modules. The analysis included healthy brain aging samples spanning young and older controls (*n* = 150) and age-filtered AD/control brain samples older than 59 years (*n* = 173). Metadata were harmonized to standardized fields, including sample identifier, age, sex, brain region, tissue, and disease status. Gene identifiers were standardized; transcript duplicates were collapsed by retaining the feature with the highest variance; genes with <80% finite values were excluded; remaining missing values were median-imputed within gene; and zero-variance genes were removed.

To ensure comparability across the aging and AD matrices, only shared genes were retained. Dataset-specific variance ranks were combined, and the top 15,000 shared genes were used as the consensus gene universe. WGCNA was performed on the transposed healthy-aging expression matrix. The soft-thresholding power was estimated using a signed-network criterion, and the primary network was constructed using a signed topological overlap matrix with networkType = “signed”, TOMType = “signed”, minModuleSize = 120, and mergeCutHeight = 0.25. Module eigengenes were calculated and ordered before association testing.

Module–trait associations were evaluated using linear models with age as a continuous variable and AD status as a binary variable. Models included available covariates where estimable, including sex, batch, brain region, tissue, and age where applicable. Noninformative or unstable predictors were removed before modeling, including invariant covariates, highly collinear predictors (Pearson *r* > 0.98), and observations with missing model variables. Two-sided coefficient tests were used to estimate module–trait association *P* values, followed by Benjamini–Hochberg FDR correction. Modules were classified as AP-like only when age- and AD-associated effects showed opposite directions at the module eigengene level. AP-Resilience modules were defined by positive age association and negative AD association, whereas AP-Vulnerability modules were defined by negative age association and positive AD association. Module-level AP scores were derived from the magnitude and direction of age and AD effects, and module-level evidence was additionally summarized using Fisher-combined *P* values. These labels define operational coexpression categories for prioritizing modules with opposite age-AD directionality.

Gene-level age and AD associations were estimated using multiple linear regression with the same covariate-aware framework where applicable. Aging-associated gene effects across brain regions were summarized by inverse variance-weighted effect estimates and Fisher-combined *P* values. Per-gene AP scores were then calculated from the direction and magnitude of age- and AD-associated effects, normalized to a 0 to 1 scale, and additionally reported as robust *z* scores and percentiles. Genes were assigned to AP-Resilience, AP-Vulnerability, or other classes according to their age/AD directionality. Within each module, intramodular connectivity was estimated as the Pearson correlation between gene expression and the corresponding module eigengene. Gene-level evidence within modules was aggregated using weighted Stouffer *Z* scores, with mean module membership/connectivity used as the weight, followed by FDR correction. The top 10 hub genes per module were exported. For prioritized AP-like modules, ego networks were constructed from the 15 highest-scoring genes per AP class using topological overlap similarity, with adjacency used as a fallback when required.

To assess robustness to WGCNA parameter choices, the primary network was retained as the main analysis and a separate parameter-sensitivity audit was performed. This audit varied soft-thresholding power (5, 6, and 7), minimum module size (80, 120, and 160 genes), and module merge cut height (0.20, 0.25, and 0.30), yielding 27 parameter settings. For each setting, modules were reclassified using strict eigengene-level AP directionality. Robustness was evaluated using AP-module counts, AP class-specific module counts, overlap of top AP genes with the primary analysis, primary-module gene-content overlap, and gene-module AP consistency. Gene-level AP scores were analyzed separately from module-level AP labels; therefore, AP-like genes located in non-AP modules were not used to relabel those modules.

### Functional enrichment of AP-derived gene sets

AP-ranked genes from WGCNA were analyzed with optional module-level summaries. Symbols were standardized to HGNC; modules were treated as categorical factors. Unless otherwise specified, only genes above a predefined AP percentile (default 90th) were retained. If module summaries were available, modules with FDR <0.05 were prioritized; otherwise, all modules were analyzed. Global and per-module sets were created for (a) all AP genes, (b) resilience-like genes, and (c) vulnerability-like genes, with minimum set size constraints (default ≥5).

To control for detectability/annotation coverage, the enrichment universe comprised unique nonmissing symbols from the aging consensus meta-analysis (minimum ≥50 genes). Human symbols were mapped to Entrez IDs with org.Hs.eg.db (mapIds; first match retained), and only mapped IDs were tested. Over-representation analyses for GO [Biological Process (BP), Cellular Component (CC), and Molecular Function (MF)] and Kyoto Encyclopedia of Genes and Genomes (KEGG) (hsa) were conducted with clusterProfiler using hypergeometric tests and Benjamini–Hochberg correction (default thresholds: nominal *P* ≤ 0.05, *q* ≤ 0.20). GO redundancy was optionally reduced with clusterProfiler::simplify using semantic similarity and p.adjust ranking statistics [37,38].

### Cell-state enrichment analysis and cell type-adjusted differential expression

To evaluate whether bulk-brain transcriptomic differences could be driven by changes in underlying cellular composition, we performed a marker-based cell-state enrichment analysis on the GSE48350 brain expression matrix. Gene-level bulk expression matrices were first harmonized to HGNC symbols, duplicate symbols were collapsed by mean expression, and sample identifiers were matched to phenotype metadata. Brain cell-state signatures were obtained from MSigDB C8 brain cell-type marker collections [35]; cell-state assignments comprised microglia, astrocyte, oligodendrocyte, endothelial, pericyte, excitatory neuron, and inhibitory neuron signatures. For each sample, cell-state enrichment scores were computed using 2 complementary approaches: single-sample rank-based scoring (singscore) and per-signature mean *z*-score aggregation across marker genes. For downstream covariate adjustment, 4 marker-based enrichment scores (microglia, astrocyte, oligodendrocyte, and endothelial) were entered as continuous covariates in linear models. Excitatory and inhibitory neuron marker scores were highly correlated with each other (Pearson *r* = 0.94) and were not entered jointly with the compact adjustment set to avoid rank deficiency and overfitting. AD differential expression was then re-estimated using limma/empirical Bayes [31] both before and after adjustment for estimated cell-state enrichment, together with available biological covariates including age, sex, and brain region. To assess robustness, we compared pre- and post-adjustment log fold changes, the overlap of significant genes, the retention of prioritized AP genes, and pathway-level consistency using Hallmark gene sets. These analyses were designed to determine whether the main AD/AP signals remained detectable after accounting for marker-based cell-state composition effects in bulk tissue; they do not establish cell-intrinsic regulation and require single-cell or spatial validation.

### External validation in independent AD cohorts

To evaluate whether the prioritized AP candidates were reproducible outside the discovery framework, we performed an independent external validation analysis in 3 AD transcriptomic cohorts: GSE118553 temporal cortex, GSE33000, and GSE5281 entorhinal cortex. Each cohort was processed using dataset-specific phenotype parsing and platform-aware probe annotation to avoid errors introduced by forced generic metadata harmonization. Expression matrices were inspected for log-scale compatibility and normalized when required; probes were mapped to HGNC gene symbols using platform annotation resources and collapsed to gene level by retaining the probe with the highest inter-sample variability. Within each validation cohort, differential expression was estimated for AD versus control samples using limma linear models with empirical Bayes moderation. Age and sex metadata were included as covariates when available and sufficiently noncollinear; otherwise, the model was restricted to the identifiable design factors and the excluded covariates were recorded in quality-control outputs. External validation used a predefined AP-directionality rule with fixed expected orientation: AP-Resilience genes were expected to show decreased expression in AD, whereas AP-Vulnerability genes were expected to show increased expression in AD. Prespecified analysis constants were applied without post hoc orientation flipping: FDR threshold = 0.05, nominal P threshold = 0.05, primary top-K = 500, secondary top-K = 1,000, minimum group *n* = 4, random seed = 42. For each cohort, we reported the number of testable AP genes, directional concordance, nominal direction-concordant support, FDR-level direction-concordant support, and FDR-level association irrespective of direction. Cross-cohort evidence was summarized using signed Z statistics aligned to the expected AP direction and combined by Stouffer’s method across available cohorts.

## Results

### Overview of age- and AD-associated transcriptomic changes

Comparative transcriptomic profiling of human dermal fibroblasts and multiple brain regions, including the entorhinal cortex (EC), hippocampus (HPC), post-central gyrus (PCG), and superior frontal gyrus (SFG), revealed widespread gene-expression alterations associated with both operational aging contrasts and AD. Ranking transcripts by log₂FC identified concordant and divergent directionality patterns within independently modeled contrasts while also exposing region-specific effects.

To visualize aging-associated genes with evidence of cross-context directional sharing, we selected genes that were significant in dermal fibroblast aging and in at least one brain-region aging contrast in the same direction (FDR < 0.05 and |log₂FC| > 0.25). Because the goal was to display directionally shared aging patterns and not a universally significant cross-tissue core, genes were not required to be significant in all 4 brain regions.

**Fig. 2.**
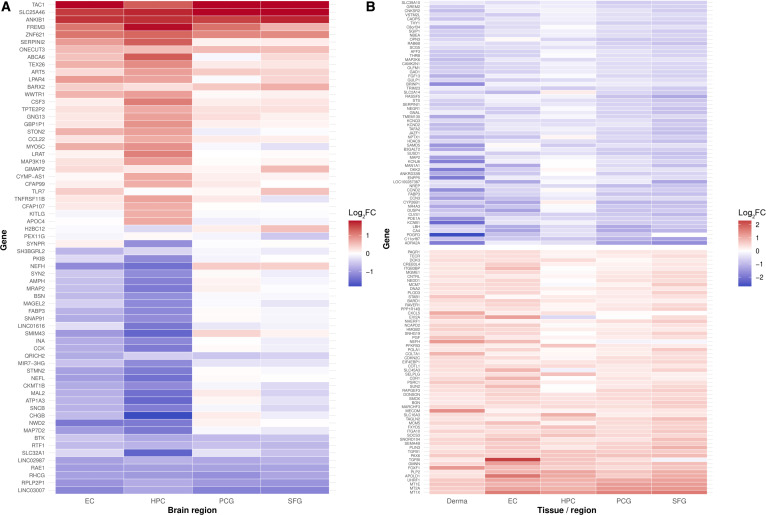
Gene-level dysregulation in AD and aging. (A) Heatmap of region-specific log_2_FC values for selected significant AD-associated genes in EC, HPC, PCG, and SFG. Genes were selected from AD versus control contrasts using FDR < 0.05 and |log_2_FC| > 0.5; rows are ordered by mean log_2_FC. Red indicates higher expression and blue indicates lower expression in AD. (B) Heatmap of log_2_FC values for direction-concordant aging-associated genes shared between dermal fibroblasts and at least one brain region. Genes were selected using FDR < 0.05 and |log_2_FC| > 0.25 in independently modeled aging contrasts. Displayed values do not imply significance in every tissue/region. Rows show shared down-regulated genes first, followed by shared up-regulated genes; blue indicates lower and red indicates higher expression with age.

In AD, cross-regional analyses of EC, HPC, PCG, and SFG demonstrated a composite architecture of shared and region-enriched dysregulation. In the discovery dataset, AD-associated up-regulated transcripts included TAC1, SLC25A46, ANKIB1, FREM3, ZNF621, SERPINI2, ONECUT3, ABCA6, ART5, LPAR4, WWTR1, and CSF3, with the strongest induction observed in EC and HPC. By contrast, transcripts such as RAE1, RHCG, RPLP2P1, PKIB, NEFH, SYN2, AMPH, MRAP2, BSN, MAGEL2, FABP3, SNAP91, INA, NEFL, ATP1A3, SNCB, CHGB, MAP7D2, and SLC32A1 were consistently down-regulated, with the largest decreases concentrated in HPC and SFG, indicating advanced disruption of neuronal and synaptic programs in these regions (Fig. 2A).

Across aging comparisons, a substantial set of transcripts decreased with age, including ADRA2A, PDGFD, CA4, LBH, KCNB1, PDE1A, DUSP4, NR4A3, CYP26B1, MAP2, KCNJ6, KCND2, KCNQ3, NEGR1, THY1, CADPS, CNKSR2, GREM2, HDAC9, JAZF1, and CCND2, supporting a broad decline in transcriptional programs spanning peripheral and central tissues. Conversely, age-increased genes, including MT1X, MT2A, MT1E, UHRF1, MCM5, MCM7, DNA2, GMNN, SOCS3, FXYD5, TAGLN2, TGFB1, TGFBI, PLIN3, MECOM, SUN2, SMOX, and DONSON, suggested activation of stress-adaptive, remodeling, and proliferative-response modules in later life (Fig. 2B).

To separate early developmental dynamics from adult age-associated signatures, we further compared dermal fibroblast transcriptomes between children (0 to 16 years) and young adults (17 to 45 years) in GSE113957. The most strongly up-regulated developmental transition genes included RGS16, HTR1F, KRT14, and CDH18, whereas prominent down-regulated genes included PTGDS, MMP12, CLEC2A, CCN5, and THBS4 (Table S2 and Fig. S1). Functional enrichment analysis of biological process terms showed dominant mitochondrial and respiratory themes, including mitochondrial electron transport [nicotinamide adenine (NADH) to ubiquinone], proton motive force-driven adenosine triphosphate (ATP) synthesis, oxidative phosphorylation, respiratory electron transport chain, aerobic respiration, cellular respiration, and energy derivation by oxidation of organic compounds (Fig. S2).

According to these GO findings, KEGG enrichment converged on mitochondrial and oxidative-stress-related pathways. The top pathways included Oxidative phosphorylation, Thermogenesis, Chemical carcinogenesis-reactive oxygen species, and Diabetic cardiomyopathy, together with multiple neurodegeneration-related pathways (AD, Parkinson’s disease, Huntington’s disease, amyotrophic lateral sclerosis, and prion disease). These categories are unified by a shared core of respiratory-chain and mitochondrial metabolism genes (Fig. S3).

### Covariate and dermal-exclusion sensitivity analyses.

In the fibroblast adult-aging covariate audit, 98 GSE113957 samples were retained for the predefined young-adult versus old-adult comparison, including 42 young-adult and 56 old samples, with 28 female and 70 male samples. Sex was available and estimable in the DESeq2 design. The primary and sex-adjusted fibroblast aging effect sizes were highly concordant genome-wide (Spearman ρ = 0.988), indicating that inclusion of sex did not materially alter the adult fibroblast aging effect-size structure. However, only 23 of 41 prioritized AP candidates were testable after feature-to-symbol mapping in this fibroblast audit, and AP class-consistent adult fibroblast directionality was observed for a subset of candidates. Therefore, the fibroblast sensitivity result was interpreted as evidence of effect-size stability after sex adjustment, not as independent fibroblast-level validation of every AP gene. In the AD brain covariate audit, 173 age-filtered GSE48350 brain samples and 21,356 genes were modeled with sex, age, and brain region included as estimable covariates. Primary and covariate-adjusted AD effect sizes were strongly concordant (Spearman ρ = 0.985). All 41 prioritized AP candidates retained the expected AD-direction sign after covariate adjustment, with FDR-level support for 38/41 candidates. Finally, in the brain-only dermal-exclusion audit, all 41 prioritized AP candidates were testable and retained the same AP class when only brain aging and AD brain meta-effects were used: 7/7 AP-Resilience genes and 34/34 AP-Vulnerability genes remained in the same-class. These results indicate that the primary AP directionality was not driven by the inclusion of dermal fibroblast aging information.

### Integrated hallmark pathway across brain aging and AD

**Fig. 3.**
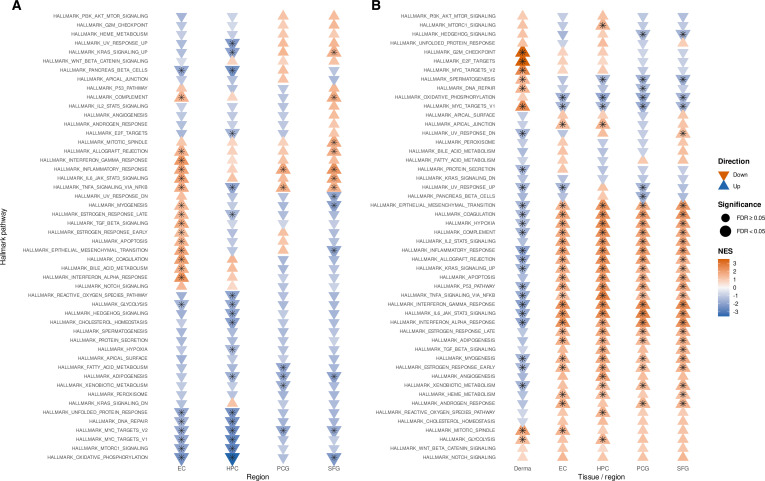
Transcriptomic Hallmark pathway enrichment in AD and aging across brain regions. (A) AD-associated Hallmark enrichment across EC, HPC, PCG, and SFG. Color indicates NES; positive NES denotes enrichment among genes up-regulated in AD, and negative NES denotes enrichment among genes down-regulated in AD. Triangle orientation indicates enrichment direction. Marker size reflects −log_10_(FDR), and asterisks mark pathways significant at FDR < 0.05 after Benjamini–Hochberg correction. (B) Aging-associated Hallmark enrichment across dermal fibroblasts, EC, HPC, PCG, and SFG. Positive NES denotes enrichment among genes that increased with age, whereas negative NES denotes enrichment among genes that decreased with age.

To characterize pathway-level remodeling in healthy aging and AD, we performed Hallmark gene set enrichment analysis (GSEA; FDR < 0.05) across 4 cortical regions—EC, HPC, PCG, and SFG—and then integrated aging- and AD-associated NESs into a unified comparative framework (Fig. 3A and B and Fig. S4). Because metabolic suppression and immune activation are well-established features of AD, these Hallmark results provide directional context for the AP framework. The key comparison was therefore not whether these pathways are present in AD, but whether their aging-associated and AD-associated directions were concordant or divergent. Across regions, both states engaged stress and immune signaling while attenuating core metabolic programs, but AD exhibited a markedly stronger inflammatory shift together with deeper suppression of mitochondrial and lipid-homeostatic networks.

In AD, the most consistently enriched Hallmark programs were inflammatory and stress-responsive pathways. These included tumor necrosis factor α (TNFα) signaling via nuclear factor κB (NF-κB), inflammatory response, and interleukin-6 (IL6)/Janus kinase (JAK)/signal transducer and activator of transcription (STAT3) signaling, accompanied by complement and interferon-α/γ response signatures. Additional enrichment of mTORC1 signaling, coagulation, KRAS signaling UP, angiogenesis, unfolded protein response, and DNA repair was observed. These programs suggested coordinated gliovascular remodeling and proteostatic pressure. In parallel, AD showed strong negative enrichment for pathways supporting neuronal energetics and cellular maintenance, including oxidative phosphorylation, fatty acid metabolism, peroxisome, and cholesterol homeostasis, together with reduced MYC targets and E2F targets, supporting impaired biosynthetic capacity and network-level metabolic failure. Although these directionality patterns were conserved across regions, their amplitudes varied, with immune pathway activation most pronounced in HPC and PCG and suppression of metabolic/bioenergetic programs strongest in EC and SFG, consistent with regional differences in vulnerability.

Healthy aging displayed a partially overlapping but more balanced Hallmark profile. Xenobiotic metabolism and coagulation were recurrently enriched, according to adaptive modulation of detoxification and vascular-associated processes, and low-grade activation of inflammatory signaling (e.g., IL6/JAK/STAT3 and TNFα/NF-κB) was also observed. However, compared with AD, immune enrichment was generally lower in magnitude and occurred in a context where metabolic and neuronal-supportive programs were comparatively better preserved. Aging-related negative enrichment was dominated by gradual reductions in oxidative phosphorylation and lipid-associated metabolism (including fatty acid and bile acid metabolism) together with selected proliferative/stress modules, with regional heterogeneity that was most evident in SFG and HPC.

To formalize the relationship between these 2 states, we constructed an antagonism map by plotting Hallmark NES for aging (*x* axis) against AD (*y* axis), which stratified pathways into 4 directional classes. A prominent (Age↑, AD↓) quadrant (AP-Resilience) was enriched for metabolic and lipid programs, including adipogenesis, glycolysis, cholesterol homeostasis, heme and fatty acid metabolism, peroxisome, and steroid hormone responses. This pattern indicates that pathways with relatively higher aging-associated enrichment showed lower enrichment in AD within this comparative framework. In contrast, immune and inflammation-related pathways largely clustered in a SharedUp class (Age↑, AD↑), showing concordant activation across aging and AD (e.g., inflammatory response, TNFα/NF-κB, IL6/JAK/STAT3, interferon, complement, coagulation, and TGF-β signaling). Oxidative phosphorylation and proteostasis-associated stress programs primarily occupied a SharedDown class (Age↓, AD↓), supporting coordinated repression in both contexts, typically with stronger suppression in AD. A smaller set of pathways displayed (Age↓, AD↑) behavior (AP-Vulnerability), indicating selective disease-associated induction against an aging-related decline. Collectively, this integrated Hallmark landscape supports a model in which healthy aging and AD share several established AD-relevant pathway themes, including inflammatory activation and metabolic impairment, but differ in the direction and relative magnitude of selected homeostatic, lipid-metabolic, mitochondrial, and proteostatic (protein homeostasis) programs. Thus, the pathway analysis provides biological context for AP-like directionality and anchors established AD mechanisms within the aging–AD directionality framework.

### Directionality mapping of aging and AD

**Fig. 4.**
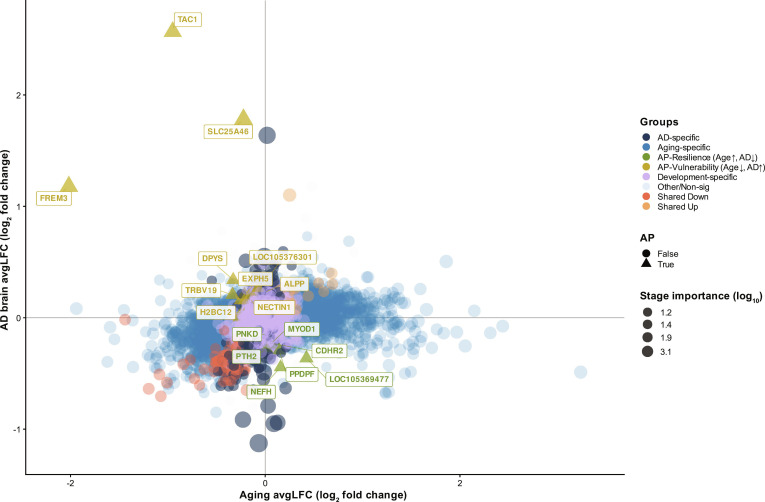
Aging versus AD gene effects across the brain. Each point represents a gene positioned by its meta-analyzed log_2_ fold change in healthy brain aging and AD. Zero reference lines indicate no directional effect. Colors show bucket-level trajectory categories, including aging-specific, AD-specific, shared, nonsignificant, and AP groups. Triangles mark strict AP genes: AP-Resilience (Age↑, AD↓) and AP-Vulnerability (Age↓, AD↑). Point size reflects the precomputed stage_importance value from the bucket-level summary table, with larger points indicating higher stage-level prioritization. Labeled genes are the top-prioritized AP candidates.

To evaluate the directionality of gene-expression changes across non-AD aging and AD, gene-level log₂FC estimates were integrated after dataset-specific differential expression modeling and projected into a 2-dimensional effect space, with the aging effect on the *x* axis and the AD effect on the *y* axis (Fig. 4). This analysis focused on relative effect direction after platform-specific modeling. The global distribution was centered close to zero, indicating that most genes showed limited combined directional shift. However, a subset of genes occupied opposite-direction quadrants, supporting the presence of directionally divergent aging–AD expression patterns within the integrated framework.

Genes in the lower-right quadrant (Age↑/AD↓) were classified as AP-Resilience-like candidates, representing transcripts that increased during non-AD aging but decreased in AD. This pattern is compatible with age-associated programs that may be attenuated or lost in disease, although it does not establish a protective or causal role. Genes in the upper-left quadrant (Age↓/AD↑) were classified as AP-Vulnerability-like candidates, representing transcripts that declined with aging but increased in AD. This directionality may reflect disease-associated reactivation, maladaptive induction, altered cellular composition, or secondary responses to neurodegenerative stress. Concordant-direction quadrants represented genes changing in the same direction during aging and AD. Shared-up genes (Age↑/AD↑) and shared-down genes (Age↓/AD↓) were interpreted as common-direction aging–AD programs. These categories were retained to distinguish divergent expression trajectories from broader aging- or disease-associated changes.

Developmental effects were evaluated separately to reduce misclassification of early-life maturation programs as late-life AP-like signals. Genes were classified across 3 transitions: development, defined as 0 to 16 versus 17 to 45 years; adult aging, defined as 17 to 45 versus >59 years; and AD, defined as AD versus age-matched controls. Development-only genes were defined as transcripts significantly altered during the developmental comparison but not detectably modulated during adult aging or AD. These genes were interpreted as early-life maturation-associated transcripts and were not prioritized as AP-like candidates. In contrast, partly developmental genes showed a developmental shift plus at least one adult-aging or AD-associated change and were therefore retained for downstream interpretation when their late-life directionality satisfied the AP-like criteria.

This directionality framework separates 3 transcriptomic patterns: development-restricted changes, concordant aging–AD changes, and opposite-direction aging–AD changes. Accordingly, AP-Resilience-like and AP-Vulnerability-like labels are used here as operational transcriptomic categories that prioritize candidate genes for further mechanistic validation. They define data-derived directionality classes and are not clinical resilience/vulnerability labels (Table 1).

**Table 1.** Expression trajectories and AP classification of genes across development, aging, and AD. The table summarizes gene expression changes during development (0 to 16 → 17 to 45 years, dermis), adult aging in dermis and brain (17 to 45 → >59 years), and AD-related change (AD versus age-matched old controls, brain). Arrows indicate direction of change (↑ increase, ↓ decrease, - no significant change). Genes were assigned to AP categories based on whether their age-related patterns in healthy adults are preserved or reversed in AD, distinguishing AP-Vulnerability (Age↓, AD↑) from AP-Resilience (Age↑, AD↓). For the complete table, see Table S3.

Gene	Development (0–16 vs. 17–45, dermis)	Adult aging (17–45 vs. >59, dermis)	Adult aging (17–45 vs. >59, brain)	AD change (AD vs. old control, brain)	Interpretation
SLC25A46	↓	↓	↓	↑	AP-Vulnerability (Age↓, AD↑)
FREM3	-	-	↓	↑	AP-Vulnerability (Age↓, AD↑)
TAC1	-	-	↓	↑	AP-Vulnerability (Age↓, AD↑)
ALPP	-	-	↓	↑	AP-Vulnerability (Age↓, AD↑)
NECTIN1	-	↑	↓	↑	AP-Vulnerability (Age↓, AD↑)
EXPH5	-	↓	↓	↑	AP-Vulnerability (Age↓, AD↑)
RFTN1	-	↓	↓	↑	AP-Vulnerability (Age↓, AD↑)
KRT9	-	-	↓	↑	AP-Vulnerability (Age↓, AD↑)
NPHP1	-	↓	↓	↑	AP-Vulnerability (Age↓, AD↑)
PKP1	-	-	↓	↑	AP-Vulnerability (Age↓, AD↑)
DCHS2	-	↓	↓	↑	AP-Vulnerability (Age↓, AD↑)
CT83	-	-	↓	↑	AP-Vulnerability (Age↓, AD↑)
AK7	-	-	↓	↑	AP-Vulnerability (Age↓, AD↑)
MYOD1	-	-	↑	↓	AP-Resilience (Age↑, AD↓)
PTH2	-	-	↑	↓	AP-Resilience (Age↑, AD↓)
NEFH	-	↑	↑	↓	AP-Resilience (Age↑, AD↓)
PPDPF	-	-	↑	↓	AP-Resilience (Age↑, AD↓)
SERPINF2	-	-	-	↓	AD-specific
CXCR6	-	-	-	↑	AD-specific
NECTIN4	-	-	-	↑	AD-specific
BTK	-	-	-	↓	AD-specific
C4A	-	-	↑	-	Aging-specific
SERPINA3	-	-	↑	-	Aging-specific
ITGB1BP2	↑	-	-	-	Development-specific
HARS2	↑	-	-	-	Development-specific
IRF6	↑	-	-	-	Development-specific

### AP landscape defined by WGCNA modules

To identify coexpression programs linked to healthy aging and AD, we performed WGCNA and evaluated module eigengene–trait relationships in the GSE48350 cohort. We then prioritized biologically informative modules for downstream interpretation and functional annotation using GO and KEGG enrichment. Within this framework, modules ME03 and ME04 emerged as key AD-associated candidates, while age-linked modules provided the complementary axis required to interpret AP dynamics.

To assess whether these WGCNA-derived patterns were sensitive to network-construction parameters, we performed a prespecified sensitivity audit across 27 settings varying soft-thresholding power, minimum module size, and module merge cut height. Strict eigengene-level AP modules remained within a narrow range of 5 to 8 modules per setting, and primary-module gene-content overlap was high overall (median best-match Jaccard = 0.955). By contrast, overlap of the top AP-ranked genes was moderate (median Jaccard = 0.465), indicating that individual gene rankings were more parameter-sensitive than the broader module architecture. We therefore used WGCNA as a coexpression contextualization and prioritization layer, with module-boundary and gene-list invariance evaluated through the sensitivity audit (Fig. S5).

### Module–trait associations with AD status

**Fig. 5.**
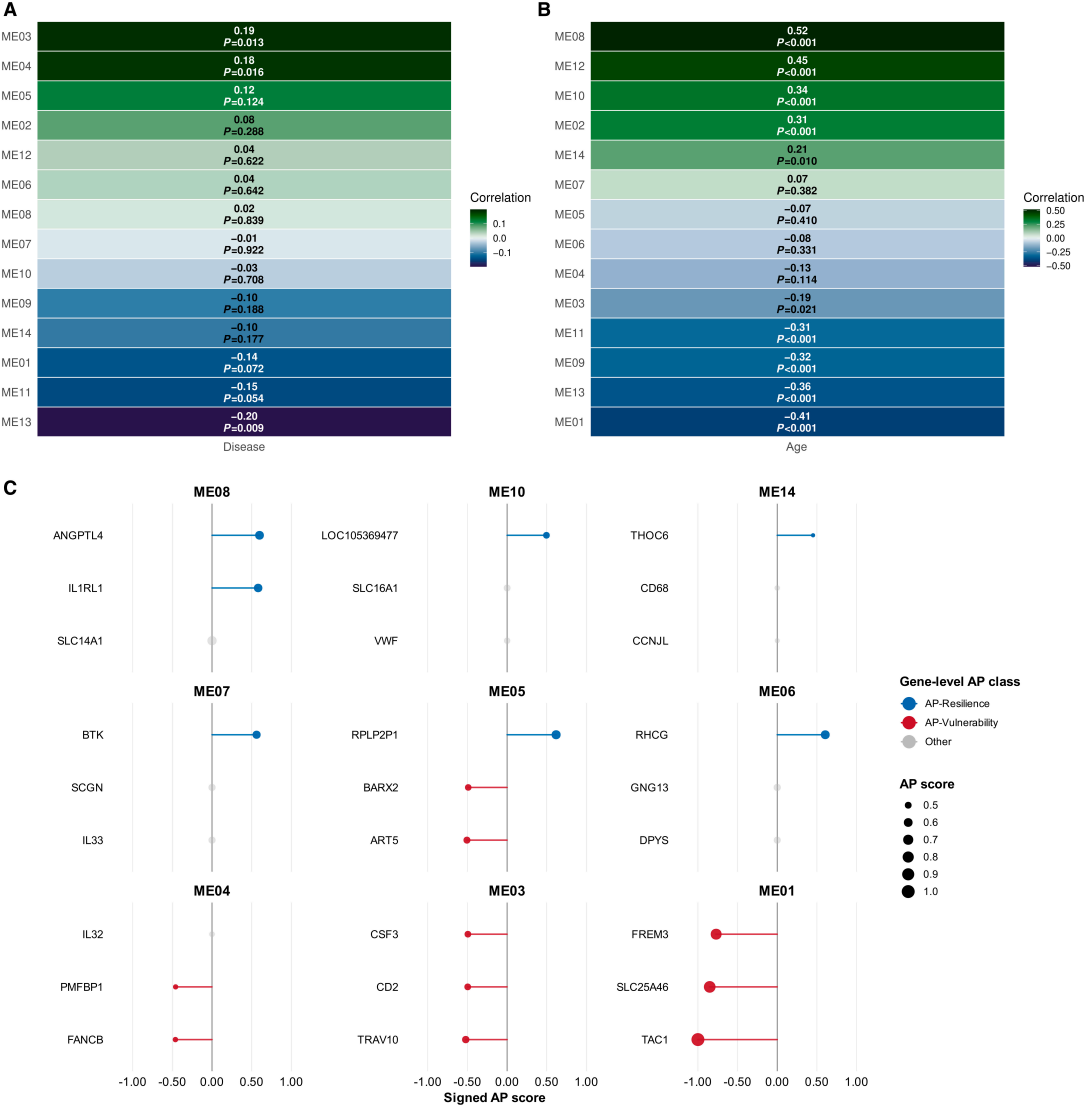
(A) Module–trait correlations in AD (GSE48350). Heatmap showing Pearson correlations between module eigengenes and AD status (*r* and *P* shown; dark green = higher in AD, dark blue = lower). (B) Module–trait correlations with age in healthy individuals (GSE48350). Heatmap showing eigengene correlations with chronological age (*r* and *P* shown; dark green = increased with age, dark blue = decreased). (C) Top antagonistically pleiotropic genes per module. Each bar represents the top 3 genes per module ranked by normalized AP score (0 to 1), reflecting the joint strength and direction of age- and AD-related associations. Colors indicate AP class; green for AP-Resilience (Age ↑/AD ↓) and orange for AP-Vulnerability (Age ↓/AD ↑). Dashed vertical lines mark the high-AP threshold (90th percentile). Modules ME01 and ME08 displayed the highest gene-level AP scores, illustrating opposing biological strategies of aging resilience and disease vulnerability within shared gene networks.

We first evaluated correlations between module eigengenes and AD status. Effect sizes were modest but directionally consistent (|*r*| ≤ 0.20), as expected for a heterogeneous late-life neurodegenerative phenotype. The strongest positive AD associations were observed for ME03 (*r* = 0.19, *P* = 0.013) and ME04 (*r* = 0.18, *P* = 0.016), whereas ME05 showed a weaker positive trend (*r* = 0.12, *P* = 0.124) (Fig. 5A). Thus, ME03/ME04 eigengene expression was higher in AD than in age-matched controls, consistent with an AP-vulnerability-like direction.

Negative AD associations were strongest for ME13 (*r* = −0.20, *P* = 0.009), followed by ME11 (*r* = −0.15, *P* = 0.054) and ME01 (*r* = −0.14, *P* = 0.072), indicating reduced eigengene expression in AD and directional compatibility with AP-resilience-like behavior. In contrast, ME02, ME06, ME08, and ME10 showed minimal AD relationships (|*r*| < 0.1), suggesting relative diagnostic stability. Among 14 tested modules, ME13, ME03, and ME04 reached nominal significance (*P* < 0.05). Overall, the pattern of ME03/ME04 up-regulation and ME13 down-regulation in AD defined a focused antagonistic module set for downstream covariate-adjusted analyses.

### Module–trait associations with chronological age in healthy individuals

We next assessed associations between module eigengenes and chronological age in healthy individuals to define normative aging trajectories. Strong positive age associations were identified for ME08 (*r* = 0.52, *P* < 0.001) and ME12 (*r* = 0.45, *P* < 0.001), followed by ME10 (*r* = 0.34, *P* < 0.001), ME02 (*r* = 0.31, *P* < 0.001), and ME14 (*r* = 0.21, *P* = 0.010) (Fig. 5B), supporting progressively activated age-related programs.

Conversely, several modules declined with age, including ME01 (*r* = −0.41, *P* < 0.001), ME13 (*r* = −0.36, *P* < 0.001), ME09 (*r* = −0.32, *P* < 0.001), and ME11 (*r* = −0.31, *P* < 0.001), indicating progressive attenuation of neuronal/synaptic maintenance-related signals. ME06, ME07, and ME05 were near neutral (|*r*| < 0.1). In total, 9 of 14 modules were age-associated at *P* < 0.05, supporting broad but structured transcriptomic remodeling during healthy aging. Joint interpretation of age and AD axes revealed directional opposition: ME01/ME11/ME13 declined with age and were further altered in AD, whereas strongly age-positive modules (notably ME08/ME12) defined a contrasting axis. Within this framework, ME03 and ME04 remained notable as AD-elevated modules with inverse directionality relative to healthy-aging trends (Table 2).

**Table 2.** Comparison of gene coexpression module–trait correlations between AD and normal aging. WGCNA module-level age-AD directionality. Module-level AP direction was evaluated using module eigengene correlations with aging and AD status. AP-Vulnerability direction was defined as Age↓/AD↑, whereas AP-Resilience direction was defined as Age↑/AD↓. Modules with the same direction in aging and AD were not considered antagonistic at the module level. Directional AP labels indicate opposite module eigengene directions and do not imply that all genes within the module are AP genes.

Module	AD correlation	Age correlation	Module-level direction	Interpretation	
ME03	+0.19; *P* = 0.013; ↑ in AD	−0.19; *P* = 0.021; ↓ with age	Age↓/AD↑	Directional AP-Vulnerability module. This is one of the strongest module-level antagonistic patterns because both aging and AD correlations support opposite directions.	
ME04	+0.18; *P* = 0.016; ↑ in AD	−0.13; *P* = 0.114; ↓ with age	Age↓/AD↑	Directional AP-Vulnerability module, moderate support. AD association is nominally significant, while the aging effect is directionally concordant but weaker.	
ME05	+0.12; *P* = 0.124; ↑ in AD	−0.07; *P* = 0.410; ↓ with age	Age↓/AD↑	Weak directional AP-Vulnerability module. Direction is consistent with AP-Vulnerability, but both correlations are modest and not statistically supported; should be interpreted cautiously.	
ME06	+0.04; *P* = 0.642; slightly ↑ in AD	−0.08; *P* = 0.331; ↓ with age	Age↓/AD↑	Weak directional AP-Vulnerability module. Included as directionally AP-like at the module eigengene level, but effect sizes are minimal; not a strong AP module.	
ME10	−0.03; *P* = 0.708; ↓ in AD	+0.34; *P* < 0.001; ↑ with age	Age↑/AD↓	Directional AP-Resilience module, aging-driven. Strong age association with weak opposite AD trend; supports AP-Resilience direction but should not be overinterpreted as disease-robust alone.	
ME14	−0.10; *P* = 0.177; ↓ in AD	+0.21; *P* = 0.010; ↑ with age	Age↑/AD↓	Directional AP-Resilience module, moderate support. Aging association is nominally significant and AD effect is directionally opposite but weaker.	
ME07	−0.01; *P* = 0.922; essentially unchanged in AD	+0.07; *P* = 0.382; slightly ↑ with age	Age↑/AD↓	Weak directional AP-Resilience module. Direction matches AP-Resilience, but both effects are minimal; should be treated as low-confidence at module level.	
ME13	−0.20; *P* = 0.009; ↓ in AD	−0.36; *P* < 0.001; ↓ with age	Age↓/AD↓	Same-direction module; not antagonistic. Shows concordant decrease in aging and AD.	
ME01	−0.14; *P* = 0.072; ↓ in AD	−0.41; *P* < 0.001; ↓ with age	Age↓/AD↓	Same-direction module; not module-level AP. Important gene-level AP candidates may occur within this module, but the module eigengene itself is not antagonistic.	
ME11	−0.15; *P* = 0.054; ↓ in AD	−0.31; *P* < 0.001; ↓ with age	Age↓/AD↓	Same-direction module; not antagonistic. Shows consistent reduction with aging and AD.	
ME09	−0.10; *P* = 0.188; ↓ in AD	−0.32; *P* < 0.001; ↓ with age	Age↓/AD↓	Same-direction module; not antagonistic. Aging association is clear; AD effect is weaker but same direction.	
ME08	+0.02; *P* = 0.839; slightly ↑ in AD	+0.52; *P* < 0.001; ↑ with age	Age↑/AD↑	Same-direction module; not antagonistic. Strong aging-associated increase with minimal same-direction AD trend.	
ME12	+0.04; *P* = 0.622; slightly ↑ in AD	+0.45; *P* < 0.001; ↑ with age	Age↑/AD↑	Same-direction module; not antagonistic. Strong aging-associated increase with minimal same-direction AD trend.	
ME02	+0.08; *P* = 0.288; ↑ in AD	+0.31; *P* < 0.001; ↑ with age	Age↑/AD↑	Same-direction module; not antagonistic. Aging and AD correlations point in the same direction.	

### Functional annotation of antagonistic modules

To characterize biological programs represented by antagonistic modules, we performed GO and KEGG enrichment analyses for ME03 and ME04 genes. Both modules converged on receptor-centered processes linking sensory signaling, immune communication, and membrane-associated regulation, indicating organized AP-like structure within these networks.

For ME03, enriched terms included sensory perception/stimulus detection, humoral immune processes, and transmembrane signaling. Molecular-function enrichment emphasized G protein-coupled receptor (GPCR)/olfactory receptor activity, cytokine-related signaling, and ion-transport functions, with additional enrichment for endopeptidase inhibitory activity and steroid/lipid-associated metabolism. This profile supports a sensory–immune axis potentially relevant to olfactory dysfunction and inflammatory remodeling in AD (Fig. S6).

ME04 showed partial overlap with ME03 but retained distinct features. Enrichment defined sensory perception together with epithelial organization, keratinization-associated programs, and wound/repair-related biology, alongside immune-response signatures. Molecular functions again implicated GPCR-linked and cytokine-interaction pathways, while cellular-component terms (including plasma membrane/epithelial-associated compartments) supported roles in interface-like immune-sensory integration (Fig. S7). Thus, ME03 and ME04 share a receptor-mediated core but also exhibit module-specific specialization.

KEGG results were concordant with GO and emphasized cytokine–receptor interaction, olfactory transduction, and hormone/lipid metabolism pathways (Fig. S8). Taken together, these findings support interconnected receptor-driven networks coupling sensory transduction with immune and metabolic regulation.

### Prioritization of top AP genes across modules

We next ranked genes using normalized AP scores (0 to 1) and visualized the top 3 genes per module by AP directionality, distinguishing AP-resilience (Age↑, AD↓) from AP-vulnerability (Age↓, AD↑) signals. We next ranked genes using normalized gene-level AP scores and visualized the top candidates within their corresponding WGCNA module facets (Fig. 5C). This analysis was used to place AP-like genes into coexpression context, not to relabel modules as AP-Resilience or AP-Vulnerability modules. Module-level AP directionality was evaluated separately using module eigengene associations with aging and AD status (Table 2), whereas Fig. 5C displays individual gene-level AP candidates.

The strongest gene-level AP-Vulnerability candidates were observed in ME01, including TAC1, SLC25A46, and FREM3. Additional AP-Vulnerability candidates appeared in ME03 and ME04, including TRAV10, CD2, CSF3, FANCB, and PMFBP1. Gene-level AP-Resilience candidates included ANGPTL4 and IL1RL1 in ME08, RHCG in ME06, BTK in ME07, LOC105369477 in ME10, and HBA1/HBA2 in ME00. Because these classifications are assigned at the gene level, the presence of AP-like genes within a module does not imply that the corresponding module eigengene satisfies the strict module-level AP criterion.

Thus, Table 2 summarizes antagonistic directionality at the module eigengene level, whereas Fig. 5C summarizes prioritized gene-level AP candidates within their corresponding WGCNA module context. These analyses are related but not identical. A module can contain individual genes with high AP scores even when the overall module eigengene follows a same-direction aging–AD pattern. Conversely, a directionally AP-like module may contain both AP-like and non-AP-like member genes. We therefore used WGCNA primarily to provide coexpression context for AP biology, with gene-level AP scoring retained as the main criterion for AP gene prioritization (Table 3).

**Table 3.** AP genes distinguishing healthy brain aging from AD. Representative AP genes from meta-analyzed age and AD log_2_ fold changes, with gene name, AP pattern (Age↑/AD↓ or Age↓/AD↑), indicator versus maker classification, and supporting evidence from aging and AD/neurodegeneration studies. Robustness was cross-checked in 3 independent AD transcriptomic cohorts: GSE118553 temporal cortex, GSE33000 prefrontal cortex, and GSE5281 entorhinal cortex (see the Independent external validation of prioritized AP genes section; Fig. S11).

Gene	Full name	AP pattern in our data*	Putative role (indicator vs. maker)	Representative evidence in aging/cognition	Representative evidence in AD/neurodegeneration	External validation GSE33000	External validation GSE118553 Temporal cortex	External validation GSE5281 Entorhinal cortex
TAC1	Tachykinin precursor 1	Age↓/AD↑ (AP-Vulnerability)	Indicator of synaptic and neuropeptidergic signaling	TAC1 is consistently age-down-regulated in human cortex, and substance-P/TAC1 signaling supports hippocampal plasticity and learning in rodent models [12,83–85]	Multi-cohort transcriptomic analyses identify TAC1 as a frontal–cortex hub gene in AD associated with synaptic function and inflammation, with altered expression in 5xFAD mice and AD-resilient vs. AD-dementia brains [52,86]	Age↓/AD↑ (AP-Vulnerability)	Age↓/AD↑ (AP-Vulnerability)	Age↓/AD↑ (AP-Vulnerability)
FREM3	FRAS1 related extracellular matrix 3	Age↓/AD↑ (AP-Vulnerability)	Indicator of cortical structural/ECM integrity	FREM3 expression decreases with age in postmortem human cortex and is associated with faster perceptual processing and altered amygdala reactivity, suggesting an “accelerated aging” molecular signature [60]	Sex- and age-dependent FREM3 alterations are reported in human brain transcriptomic studies that also describe AD-related differences, supporting its role as an ECM-linked vulnerability marker [60]	AP-Vulnerability (Age↓, AD↑)	Age↓/AD↑ (AP-Vulnerability)	Age↓/AD↑ (AP-Vulnerability)
SLC25A46	Solute carrier family 25 member 46	Age↓/AD↑ (AP-Vulnerability)	Indicator/effector of mitochondrial dynamics	SLC25A46 participates in mitochondrial fission–fusion and cristae maintenance; loss of function compromises mitochondrial respiration and energy production [87]	Mouse and human genetic studies show that SLC25A46 mutations cause early-onset neurodegeneration with axonal pathology and brain atrophy, consistent with our finding that reduced SLC25A46 accompanies vulnerability to AD-like pathology [87]	AP-Vulnerability (Age↓, AD↑)	Age↓/AD↑ (AP-Vulnerability)	Age↓/AD↑ (AP-Vulnerability)
PPDPF	Pancreatic progenitor cell differentiation and proliferation factor	Age↑/AD↓ (AP-Resilience)	Indicator candidate (cell-state/glial-metabolic resilience; low specificity)	In analyses contrasting symptomatic vs. asymptomatic individuals with AD neuropathology, PPDPF is among the significant DE genes and its expression associates with cognitive performance domains (memory/language) [88]	PPDPF appears in AD network/coexpression “rewiring” contexts across brain regions, consistent with disease-state network shifts [89]	Age↑/AD↓ (AP-Resilience)	Age↑/AD↓ (AP-Resilience)	Age↑/AD↓ (AP-Resilience)
PTRH2 (listed as “PTH2”)	Peptidyl-tRNA hydrolase 2	Age↑/AD↓ (AP-Resilience)	Indicator/effector of mitochondrial quality control and cell-survival signaling	PTRH2 is a conserved mitochondrial protein; mechanistic work supports roles in mitochondrial bioenergetics/signaling that align with “cell survival/stress resilience” biology [90]	PTRH2 mutations cause multisystem neurologic disease with neurodegeneration features (neurodevelopmental/neurodegenerative overlap); mitochondrial quality control is a core axis in neurodegeneration [91–93]	Age↑/AD↓ (AP-Resilience)	Age↑/AD↓ (AP-Resilience)	Age↑/AD↓ (AP-Resilience)
NEFH	Neurofilament heavy chain	Age↑/AD↓ (AP-Resilience)	Marker of axonal/neuronal structural integrity	In large control cohorts, phosphorylated neurofilament heavy chain (pNfH; NEFH-derived axonal injury marker) shows a strong age effect, with higher levels at older ages, consistent with an aging-linked increase in background axonal injury burden [94]	In human AD brain spatial transcriptomics focusing on plaque–glia microenvironments, regions showing greater neuronal compromise exhibit lower expression of neuronal/axonal genes including NEFH (and NEFM), consistent with AD-associated NEFH↓ at the transcript level in vulnerable tissue contexts [95]	Age↑/AD↓ (AP-Resilience)	Age↑/AD↓ (AP-Resilience)	Age↑/AD↓ (AP-Resilience)

### TF activity antagonism between healthy aging and AD

To explore candidate upstream regulatory programs associated with AP-like gene-expression patterns, TF activity was inferred using DoRothEA regulons in the genome-wide mode. Each TF was projected according to its NES in non-AD aging and AD, generating a 2-dimensional TF directionality map (Fig. S9). This analysis served as a regulatory annotation layer for prioritizing candidate upstream programs.

The TF map separated regulators into 4 directional classes. Shared-up TFs showed positive inferred activity in both aging and AD and included immune-associated regulators such as STAT1, STAT2, and SPI1/PU.1. This pattern is consistent with inflammatory transcriptional programs that are detectable across late-life and disease-associated states. Conversely, shared-down TFs showed reduced inferred activity in both conditions and included FOXP1, BHLHE40, HOXB13, RFX2, ZEB1, ZNF24, MYC, and VDR, suggesting common attenuation of selected regulatory programs across aging and AD.

Opposite-direction TFs were interpreted as AP-like regulatory candidates. The AP-Vulnerability-like quadrant contained TFs with lower inferred activity in non-AD aging but higher inferred activity in AD, including HNF1A, ELF3, CDX2, TCF7, ETS1, ONECUT1, FOXM1, SPIB, DUX4, NFATC2, PRDM14, CREB1, and NR5A1. This pattern may reflect AD-associated induction of lineage, immune, or stress-responsive regulatory programs, although these inferences require experimental validation. In contrast, the AP-Resilience-like quadrant contained TFs with higher inferred activity in non-AD aging but lower inferred activity in AD, including PPARG, TEAD4, MXD4, ATF4, NFE2L2, HIF1A, KLF6, KLF1, LYL1, POU2F2, ARID3A, and RFX5. Several of these regulators are linked to metabolic, oxidative-stress, hypoxia-response, and homeostatic transcriptional programs, suggesting that selected adaptive regulatory signatures observed in non-AD aging may be attenuated in AD.

Overall, the TF-level analysis supported the gene- and pathway-level directionality results by identifying regulatory signatures with either concordant or opposite inferred activity between non-AD aging and AD. These TF patterns are best interpreted as hypothesis-generating regulatory associations that nominate candidate upstream programs for future mechanistic testing.

### Cell state-adjusted robustness of AD-associated AP signals

Because the GSE48350 brain dataset was generated from bulk tissue, we evaluated whether the observed AD-associated and AP-like transcriptomic patterns could be primarily attributable to differences in estimated cellular composition. We performed a marker-based brain cell-state enrichment analysis using curated glial, neuronal, vascular, and oligodendroglial signatures, and incorporated selected enrichment scores into cell-state-adjusted linear models. This analysis was designed as a cell-state-aware sensitivity analysis that complements the primary bulk-tissue differential expression framework.

AD and control samples showed differences in estimated cell-state enrichment across brain regions, consistent with the expected contribution of bulk cellular composition to AD-associated expression profiles (Fig. S10A). Cell-state scores were also correlated with each other, most prominently between excitatory and inhibitory neuronal signatures (Pearson *r* = 0.94), supporting the use of a compact adjustment model to reduce multicollinearity and rank deficiency (Fig. S10B). Accordingly, microglia, astrocyte, oligodendrocyte, and endothelial scores were included as continuous covariates in the adjusted AD differential expression model, together with available biological covariates.

After cell-state adjustment, global AD effect-size estimates remained highly concordant with the unadjusted model (Spearman ρ = 0.914; Pearson *r* = 0.934; Fig. S10C). However, the number of FDR-significant AD-associated genes decreased from 8,096 to 1,437, with 1,366 genes significant in both models. This attenuation indicates that a substantial component of the AD bulk-brain signal is composition-sensitive. Nevertheless, prioritized AP candidates were not eliminated by the adjustment: All 41 of 41 testable AP candidates retained their expected AD-associated direction, and 26 of 41 retained FDR-level support in the adjusted model (Fig. S10D). Pathway-level comparisons similarly showed that the broader contrast between inflammatory activation and attenuation of metabolic/homeostatic programs remained detectable after cell-state adjustment.

Together, these results suggest that estimated cellular composition contributes to the observed AD transcriptomic signal but does not fully account for the AP-like directionality of prioritized candidates. Because marker-based adjustment cannot distinguish cell-intrinsic regulation from changes in cellular abundance or state, these findings support the persistence of selected AP-like signals after accounting for major estimated cell-state axes and motivate single-cell or spatial validation.

### Independent external validation of prioritized AP genes

We then evaluated prioritized AP candidates in 3 independent AD transcriptomic cohorts: GSE118553 temporal cortex, GSE33000, and GSE5281 entorhinal cortex. External validation was performed using a predefined AP-class directionality rule, with AP-Resilience genes expected to decrease in AD and AP-Vulnerability genes expected to increase in AD. Across cohorts, 33, 26, and 40 AP genes were testable, respectively. Directionally concordant AD effects were observed for 17 of 33 genes in GSE118553, 12 of 26 genes in GSE33000, and 26 of 40 genes in GSE5281, corresponding to 52%, 46%, and 65% of testable AP genes. Nominal-*P* direction-concordant support (*P* < 0.05 with the AP-expected sign) was detected for 3, 7, and 7 of the key vulnerability genes, respectively, whereas FDR-level (adj. *P* < 0.05) direction-concordant support was more selective, involving 2, 5, and 3 genes, respectively. We additionally report FDR-significant any-direction support (*n* = 9, 16, and 3 genes) as a broad-association reference; this metric is not interpreted as AP-directional replication. Thus, the external datasets provided partial independent support for a reproducible subset of AP candidates.

Cross-cohort meta-analysis prioritized several recurrently supported AP genes. External support varied by gene. Three candidates met the strictest criterion of directional concordance in 3 of 3 cohorts plus FDR-level direction-concordant signal in ≥2 cohorts: PNKD and NEFH (AP-Resilience) and NPHP1 (AP-Vulnerability). KRT9 and PPT2 (AP-Vulnerability) and PPDPF (AP-Resilience) showed FDR-level direction-concordant support in one cohort with consistent directional sign in additional cohorts. TAC1 and RFTN1 (AP-Vulnerability) showed directional concordance in 2 of 2 testable cohorts but did not reach FDR-level direction-concordant significance externally; their inclusion as recurrent candidate genes is based on directional reproducibility. FREM3 and SLC25A46 (AP-Vulnerability) were testable in only one external cohort each (GSE5281) and should therefore be regarded as not yet externally validated. Importantly, FDR-level association irrespective of direction was not treated as AP replication, allowing broad AD association to be separated from AP-directional reproducibility. A parallel audit of established AD-risk genes, including APOE, ABCA7, BIN1, SORL1, TREM2, CLU, PICALM, and CR1, showed heterogeneous cohort-specific expression patterns, supporting their use as contextual AD-risk markers instead of forced AP-class members. Collectively, these results provide independent external support for selected AP candidates while indicating that replication strength is gene, region, platform, and cohort dependent (Fig. S11).

## Discussion

Our integrative transcriptomic analysis suggests that selected aging and AD-associated transcriptomic programs show opposite-direction patterns, consistent with an operational AP-like framework. In healthy aging, pathways related to metabolism, lipid handling, and antioxidant defense were relatively preserved or increased, whereas AD was characterized by broad attenuation of these programs together with stronger immune-inflammatory activation. This directional contrast is corroborating prior reports that aging brains can retain adaptive oxidative-stress and metabolic responses [12,13], while AD is associated with mitochondrial dysfunction, synaptic vulnerability, and microglial reactivity [14,16]. Whole-genome sequencing-based pathway prioritization further supports the centrality of immune-inflammatory, metabolic, synaptic, protein-homeostatic, epigenetic, and cell-cycle mechanisms in AD biology [17].

A key contextual point is that the primary studies underlying our input datasets were not designed to test AP explicitly, yet their original conclusions support several elements of our integrated model. The dermal fibroblast RNA-seq cohort we used has been reported as a robust substrate for chronological age prediction and for capturing aging-related transcriptomic remodeling in peripheral cells [24]. Likewise, influential brain transcriptome studies emphasized that aging effects are region- and sex-dependent, and that synaptic/neuronal programs are broadly down-regulated across aging and further impacted in AD, particularly in vulnerable limbic regions [25,26]. Building on this foundation, our contribution is not to redefine those datasets’ main messages but to connect them under a unified directionality framework, explicitly separating “shared” aging/AD changes from inversions that distinguish adaptive aging from neurodegeneration.

This interpretation provides new insights for potential therapeutic targets and strategies and is different from the accelerated-aging model of AD. Several transcriptomic studies support substantial overlap between normal brain aging and AD: Age-associated expression changes often occur in the same direction as AD-associated changes, and a subset of cognitively or clinically normal aged individuals can show AD-like transcriptomic profiles, particularly in hippocampal and cortical regions [39,40]. Sex- and region-dependent analyses have also shown that some age-related prefrontal transcriptional programs resemble AD-related changes, supporting the view that accelerated or exaggerated aging can contribute to AD susceptibility [41].

However, recent cell-state and systems-level analyses indicate that AD is not fully reducible to a linear acceleration of normal aging. A data-driven reconstruction of brain cellular communities identified a trajectory leading to AD that was distinct from other aging-related effects, emphasizing disease-specific cellular-state organization beyond chronological aging alone [42].

Our findings are consistent with this reconciled model: AD shares inflammatory, metabolic, synaptic, and cellular-composition features with aging, but selected genes and pathways show disease-specific amplification, attenuation, or opposite-direction behavior to healthy aging. Thus, the AP framework does not argue for a universal acceleration of aging in AD; rather, it identifies a subset of transcriptomic programs and, ultimately, candidate future therapeutic target genes, in which adaptive or homeostatic aging-associated responses may be lost, reversed, or overwhelmed during neurodegeneration.

TF activity inference further clarified these trajectories. DoRothEA-based estimates suggested relative activation of PPARG, NFE2L2/NRF2, and TEAD4 in aging, with opposite trends in AD, supporting evidence linking PPARG and NRF2 to mitochondrial quality control and inflammatory restraint [43]. Conversely, AD-associated increases in FOXM1, HNF1A, and ELF3 related regulatory activity were compatible with prior observations of aberrant cell-cycle/developmental signaling in neurodegeneration [44–46]. In addition, shared-up immune regulators such as STAT1, STAT2, and SPI1 likely contribute to microglial-state remodeling across conditions, in line with IFN-I/STAT signaling in AD models and SPI1/PU.1 involvement in AD-risk regulatory programs [47,48]. The enrichment of pleiotropic expression quantitative trait loci (eQTLs) in enhancer and TF binding contexts provides an orthogonal genetic rationale for interpreting directionally divergent TF activity as a plausible regulatory substrate of pleiotropic expression programs [4]. These TF-level patterns provide a plausible regulatory layer linking pathway-level directionality to disease-state transition. Importantly, DoRothEA/decoupleR-based TF activity is inferred from downstream target-gene expression and is therefore hypothesis-generating rather than causal; the regulators discussed above are presented as candidate regulators and require perturbational validation in human induced pluripotent stem cell (iPSC)-derived or in vivo systems before firm and clear mechanistic conclusions can be drawn.

Gene-level AP candidates offered complementary biological anchors. TAC1, FREM3, and SLC25A46 defined distinct biological domains, neuropeptide signaling, extracellular matrix/cortical architecture, and mitochondrial structure, each potentially relevant to aging–AD divergence. TAC1 biology supports context-dependent roles in stress and affective regulation [49–51], and network-level AD studies place TAC1 near synaptic-inflammatory interfaces [52]. SLC25A46, implicated in cristae organization, is compatible with mitochondrial maintenance hypotheses in aging and failure modes in AD [53]. FREM3, a layer-associated extracellular matrix gene, may mark cortical/extracellular-matrix remodeling programs that differ between aging and AD contexts [54]. Although these genes are not necessarily top GWAS loci, they exemplify context-dependent pleiotropy detectable through cross-tissue transcriptomic integration [7,8].

We also note that several Hallmark pathways showed opposite-direction aging effects between dermal fibroblasts and cortical regions, while the brain-only AP sensitivity audit retained AP classes for all prioritized candidates (41 of 41 after exclusion of dermal aging). This pattern supports tissue context-specific aging biology and emphasizes that brain-specific conclusions should be grounded in the brain-derived contrasts. Cross-tissue TF activity inference is similarly presented as hypothesis-generating; conserved regulator-level patterns across fibroblast and brain contexts nominate candidate programs for follow-up in tissue-appropriate systems.

The behavioral relevance of these candidates should be interpreted cautiously, but remains biologically suggestive. TAC1-related circuits have been linked to stress and addiction phenotypes [55–58], SLC25A46 to mitochondrial stress responses [59], and FREM3 to mood/cognitive traits [60]. Cross-trait analysis of smoking and facial aging has implicated ferroptosis-related IREB2 as a molecular link between environmental exposure, senescence-associated phenotypes, and immune/tumor biology, providing an example of how lifestyle factors may interact with aging-associated molecular programs [61]. Broader analyses of neuropsychiatric and digestive disorders further suggest that genetic and microbial networks can bridge brain-related phenotypes with systemic physiology through immune and synaptic pathways [62]. The observation that genes disrupted in mental disorders can be enriched for intrinsically disordered protein features also highlights the potential importance of structurally flexible proteins in neuropsychiatric vulnerability and transcriptomic network behavior [63]. Taken together, these associations are consistent with the possibility that environmental exposures and lifestyle factors interact with transcriptomic plasticity to shape late-life brain resilience, although causal pathways cannot be inferred from the present design.

From a translational perspective, our findings prioritize directionally informed targets over broad pathway suppression. Approaches that reinforce mitochondrial and lipid homeostasis (e.g., NRF2/PPARG-related programs or peroxisomal support) may be relevant to prodromal resilience, while selective calibration of complement/interferon activity may better preserve beneficial immune tone than nonspecific immunosuppression [16,43,64,65]. Network pharmacology and experimental validation studies targeting endoplasmic-reticulum stress in AD models provide a complementary example of how pathway-centered computational prioritization can nominate candidate therapeutic mechanisms for neurodegeneration [66]. Pharmacological induction models combining aluminum chloride and d-galactose in zebrafish further illustrate how aging-like and AD-like phenotypes can be experimentally coupled to cognitive deficits for mechanistic and therapeutic screening [67]. In the same vein, pathway-directional markers that increase in healthy aging but decline in AD could improve risk stratification and staging frameworks [1].

Cross-cohort integration is vulnerable to cellular-composition differences, post-mortem effects, and residual technical heterogeneity. While meta-analytic aggregation and network-based approaches reduce noise and improve robustness [36,68], single-cell and spatially resolved validation will be important to localize these signals to specific cell states and circuits. The dependence of bulk-transcriptomic deconvolution on tissue- and blood-derived cell reference matrices underscores the need to evaluate cell-composition estimates carefully when interpreting mixed-tissue aging and disease datasets [69]. Sample-level aggregation methods for single-nucleus AD classification further demonstrate how cell-resolved data can be leveraged to connect disease status with specific cell types and genes, complementing bulk-tissue directionality analyses [70].

One notable exception is SERPINA3, whose direction in our analysis differs from the common AD up-regulation trend reported in many studies. This may be explained by sex, age, and region-stratified effects (including stronger age correlation in nondemented controls and male-specific AD elevation in a multi-dataset microarray analysis), together with bulk-tissue cellular-composition differences that strongly affect glial markers [71]. Functional perturbation of candidate axes (e.g., PPARG/NRF2 and FOXM1/ELF3) in human iPSC-derived systems and in vivo models, along with longitudinal multi-omic sampling across tissues, will be necessary to test causality and temporal ordering [7,8].

Multiple independent transcriptomic efforts support the same pathway-level split we observe between healthy aging and AD. Large-scale network and meta-analytic studies of aged human cortex consistently report that AD is dominated by up-regulated innate-immune/microglial modules (complement, antigen presentation, inflammatory signaling) alongside down-regulation of neuronal/synaptic and energy-metabolic programs, including mitochondrial/oxidative phosphorylation pathways [72–74]. This immune-metabolic contrast is also evident at cellular resolution: Single-nucleus and single-cell atlases repeatedly identify AD-associated microglial/astrocytic activation states and inflammatory regulators, while neuronal populations show reduced expression of functional and bioenergetic programs across disease severity and across brain regions [75,76]. Finally, dedicated reviews and focused analyses emphasize that mitochondrial dysfunction and broader metabolic impairment are core components of AD pathophysiology, and that age-related immunometabolic shifts (including lipid-handling pathways in microglia) can become more pronounced and maladaptive in AD; providing literature support for our finding that aging shows comparatively greater preservation of metabolic/mitochondrial/lipid-homeostatic programs, whereas AD shows stronger suppression of these programs coupled to heightened inflammatory activity [77,78].

A broader consideration is that AP may operate bidirectionally. In addition to early-life beneficial programs with late-life liabilities, some late-life-protective programs may carry earlier trade-offs. Evolutionary-genetic evidence supports complex reproduction-lifespan trade-off structures [19,79], and emerging work suggests that resilience-associated late effects may be coupled to early costs in specific contexts [80,81]. Within this perspective, our AP-defined candidates provide a practical framework for testing how age-dependent benefits and liabilities are partitioned across development, aging, and AD, although they do not by themselves establish clinical resilience or resistance phenotypes.

This distinction is important because conventional AD transcriptomic analyses often prioritize dominant disease-associated signals, including inflammatory activation, synaptic loss, and metabolic dysfunction. While these signatures are biologically important, they do not by themselves distinguish whether a pathway reflects a continuation of aging, a disease-specific amplification, or a reversal of an aging-associated program. The AP framework adds this directional layer by asking whether a gene, module, pathway, or inferred regulator changes in the same or opposite direction across healthy aging and AD.

## Conclusions

A key advantage of the unified AP framework is its ability to highlight biologically consequential direction reversals. Such reversals are easily missed by conventional analyses focused on single contrasts (e.g., AD versus control) or on main effects of age in isolation. Standard differential expression or analysis of variance (ANOVA)-style summaries tend to prioritize genes with the largest absolute change in one condition, even if those genes move in the same direction in aging and AD (shared effects). In contrast, AP scoring explicitly rewards genes and regulators whose trajectories diverge between healthy aging and AD (Age↑/AD↓ or Age↓/AD↑), thereby enriching for candidates that may reflect “failed compensation”, maladaptive reactivation, or resilience-loss mechanisms. This is particularly relevant in heterogeneous late-life brain tissue where strong shared signals (e.g., immune activation and synaptic decline) can dominate ranking lists and obscure smaller but directionally informative signatures.

Our results support a model in which AD involves both shared aging-associated features and selective divergence from healthy-aging transcriptomic programs. Metabolic impairment and immune activation are established components of AD biology; here, their relevance lies in how they are positioned within aging–AD directionality maps. Directionality-based AP candidates, particularly TAC1, FREM3, and SLC25A46, provide testable markers of aging–AD divergence and candidate anchors for future mechanistic and translational studies. This AP-oriented framework may help prioritize genes and regulatory programs whose late-life trajectories distinguish preserved aging from neurodegenerative disease [82].

## Data Availability

https://github.com/salihoglu/Alzheimer_AP

## References

[1] Livingston G, Huntley J, Liu KY, Costafreda SG, Selbæk G, Alladi S, Ames D, Banerjee S, Burns A, Brayne C, et al. Dementia prevention, intervention, and care: 2024 report of the lancet standing commission. Lancet. 2024;404(10452):572–628. 39096926 10.1016/S0140-6736(24)01296-0

[2] Hou Y, Dan X, Babbar M, Wei Y, Hasselbalch SG, Croteau DL, Bohr VA. Ageing as a risk factor for neurodegenerative disease. Nat Rev Neurol. 2019;15(10):565–581. 31501588 10.1038/s41582-019-0244-7

[3] Onisiforou A, Christodoulou CC, Zamba-Papanicolaou E, Zanos P, Georgiou P. Transcriptomic analysis reveals sex-specific patterns in the hippocampus in Alzheimer’s disease. Front Endocrinol. 2024;15:1345498. 10.3389/fendo.2024.1345498 PMC11058985 38689734

[4] González A, Paul P. Pleiotropic expression quantitative trait loci are enriched in enhancers and transcription factor binding sites and impact more genes. Comput Struct Biotechnol J. 2024;23:4260–4270. 39669750 10.1016/j.csbj.2024.11.019 PMC11635986

[5] Xiong J, Xu T, Wang Z, Huang Y, Zhang S, Yang G, Yang J, Gao S, Wang T, Jian X, et al. The hidden genetic and microbial networks connecting neuropsychiatric and digestive disorders. Comput Struct Biotechnol J. 2025;27:3114–3126. 40703095 10.1016/j.csbj.2025.07.029 PMC12284710

[6] Williams GC. Pleiotropy, natural selection, and the evolution of senescence: *Evolution* 11, 398–411 (1957). *Sci Aging Knowl Environ*. 2001;2001(1):cp13.

[7] Yamamoto R, Chung R, Vazquez JM, Sheng H, Steinberg PL, Ioannidis NM, Sudmant PH. Tissue-specific impacts of aging and genetics on gene expression patterns in humans. Nat Commun. 2022;13(1):5803. 36192477 10.1038/s41467-022-33509-0 PMC9530233

[8] Consortium Gte. The GTEx consortium atlas of genetic regulatory effects across human tissues. Science. 2020;369(6509):1318–1330. 32913098 10.1126/science.aaz1776 PMC7737656

[9] Xi D, Cui D, Zhang M, Zhang J, Shang M, Guo L, Han J, Du L. Identification of genetic basis of brain imaging by group sparse multi-task learning leveraging summary statistics. Comput Struct Biotechnol J. 2024;23:3288–3299. 39296810 10.1016/j.csbj.2024.08.027 PMC11409045

[10] Lu T, Pan Y, Kao SY, Li C, Kohane I, Chan J, Yankner BA. Gene regulation and DNA damage in the ageing human brain. Nature. 2004;429(6994):883–891. 15190254 10.1038/nature02661

[11] Chesler EJ, Lu L, Wang J, Williams RW, Manly KF. WebQTL: Rapid exploratory analysis of gene expression and genetic networks for brain and behavior. Nat Neurosci. 2004;7(5):485–486. 15114364 10.1038/nn0504-485

[12] Loerch PM, Lu T, Dakin KA, Vann JM, Isaacs A, Geula C, Wang J, Pan Y, Gabuzda DH, Li C, et al. Evolution of the aging brain transcriptome and synaptic regulation. PLOS ONE. 2008;3(10): Article e3329. 18830410 10.1371/journal.pone.0003329 PMC2553198

[13] Soreq L, Rose J, Soreq E, Hardy J, Trabzuni D, Cookson MR, Smith C, Ryten M, Patani R, Ule J. Major shifts in glial regional identity are a transcriptional hallmark of human brain aging. Cell Rep. 2017;18(2):557–570. 28076797 10.1016/j.celrep.2016.12.011 PMC5263238

[14] De Strooper B, Karran E. The cellular phase of Alzheimer’s disease. Cell. 2016;164(4):603–615. 26871627 10.1016/j.cell.2015.12.056

[15] Bhatia S, Rawal R, Sharma P, Singh T, Singh M, Singh V. Mitochondrial dysfunction in Alzheimer’s disease: Opportunities for drug development. Curr Neuropharmacol. 2022;20(4):675–692. 33998995 10.2174/1570159X19666210517114016 PMC9878959

[16] Hong S, Beja-Glasser VF, Nfonoyim BM, Frouin A, Li S, Ramakrishnan S, Merry KM, Shi Q, Rosenthal A, Barres BA, et al. Complement and microglia mediate early synapse loss in Alzheimer mouse models. Science. 2016;352(6286):712–716. 27033548 10.1126/science.aad8373 PMC5094372

[17] Wang Y, Liu T, He Y, Tang Y, Tan P, Huang L, Huang D, Wen T, Shao L, Wang J, et al. Identifying Alzheimer’s disease-related pathways based on whole-genome sequencing data. Comput Struct Biotechnol J. 2025;27:4132–4144. 41050469 10.1016/j.csbj.2025.09.013 PMC12495422

[18] Austad SN, Hoffman JM. Is antagonistic pleiotropy ubiquitous in aging biology? Evol Med Public Health. 2018;2018(1):287–294. 30524730 10.1093/emph/eoy033 PMC6276058

[19] Long E, Zhang J. Evidence for the role of selection for reproductively advantageous alleles in human aging. Sci Adv. 2023;9(49): Article eadh4990. 38064565 10.1126/sciadv.adh4990 PMC10708185

[20] Campisi J. Aging, cellular senescence, and cancer. Annu Rev Physiol. 2013;75(1):685–705. 23140366 10.1146/annurev-physiol-030212-183653 PMC4166529

[21] Palmer D, Fabris F, Doherty A, Freitas AA, de Magalhães JP. Ageing transcriptome meta-analysis reveals similarities and differences between key mammalian tissues. Aging. 2021;13(3):3313. 33611312 10.18632/aging.202648 PMC7906136

[22] Zeng L, Yang J, Peng S, Zhu J, Zhang B, Suh Y, Tu Z. Transcriptome analysis reveals the difference between “healthy” and “common” aging and their connection with age-related diseases. Aging Cell. 2020;19(3): Article e13121. 32077223 10.1111/acel.13121 PMC7059150

[23] Lopez-Otin C, Blasco MA, Partridge L, Serrano M, Kroemer G. The hallmarks of aging. Cell. 2013;153(6):1194–1217. 23746838 10.1016/j.cell.2013.05.039 PMC3836174

[24] Fleischer JG, Schulte R, Tsai HH, Tyagi S, Ibarra A, Shokhirev MN, Huang L, Hetzer MW, Navlakha S. Predicting age from the transcriptome of human dermal fibroblasts. Genome Biol. 2018;19(1):221. 30567591 10.1186/s13059-018-1599-6 PMC6300908

[25] Berchtold NC, Cribbs DH, Coleman PD, Rogers J, Head E, Kim R, Beach T, Miller C, Troncoso J, Trojanowski JQ, et al. Gene expression changes in the course of normal brain aging are sexually dimorphic. Proc Natl Acad Sci USA. 2008;105(40):15605–15610. 18832152 10.1073/pnas.0806883105 PMC2563070

[26] Berchtold NC, Coleman PD, Cribbs DH, Rogers J, Gillen DL, Cotman CW. Synaptic genes are extensively downregulated across multiple brain regions in normal human aging and Alzheimer’s disease. Neurobiol Aging. 2013;34(6):1653–1661. 23273601 10.1016/j.neurobiolaging.2012.11.024 PMC4022280

[27] Salihoglu R, Balkenhol J, Dandekar G, Liang C, Dandekar T, Bencurova E. Cat-E: A comprehensive web tool for exploring cancer targeting strategies. Comput Struct Biotechnol J. 2024;23:1376–1386. 38596315 10.1016/j.csbj.2024.03.024 PMC11001601

[28] World Health Organization. Ageing and health. 2025. https://www.who.int/news-room/fact-sheets/detail/ageing-and-health

[29] Huber W, Carey VJ, Gentleman R, Anders S, Carlson M, Carvalho BS, Bravo HC, Davis S, Gatto L, Girke T, et al. Orchestrating high-throughput genomic analysis with Bioconductor. Nat Methods. 2015;12(2):115–121. 25633503 10.1038/nmeth.3252 PMC4509590

[30] Davis S, Meltzer PS. GEOquery: A bridge between the gene expression omnibus (GEO) and BioConductor. Bioinformatics. 2007;23(14):1846–1847. 17496320 10.1093/bioinformatics/btm254

[31] Ritchie ME, Phipson B, Wu D, Hu Y, Law CW, Shi W, Smyth GK. Limma powers differential expression analyses for RNA-sequencing and microarray studies. Nucleic Acids Res. 2015;43(7):e47. 25605792 10.1093/nar/gkv007 PMC4402510

[32] Love MI, Huber W, Anders S. Moderated estimation of fold change and dispersion for RNA-seq data with DESeq2. Genome Biol. 2014;15(12):550. 25516281 10.1186/s13059-014-0550-8 PMC4302049

[33] Pagès H, Carlson M, Falcon S, Li N. AnnotationDbi: Manipulation of SQLite-based annotations in Bioconductor. 2025. https://bioconductor.org/packages/AnnotationDbi doi:10.18129/B9.bioc.AnnotationDbi

[34] Xu S, Hu E, Cai Y, Xie Z, Luo X, Zhan L, Tang W, Wang Q, Liu B, Wang R, et al. Using clusterProfiler to characterize multiomics data. Nat Protoc. 2024;19(11):3292–3320. 39019974 10.1038/s41596-024-01020-z

[35] Bhuva D, Smyth G, Garnham A. msigdb: An ExperimentHub package for the molecular signatures database (MSigDB). R package version 1.20.0. https://bioconductor.org/packages/msigdb

[36] Langfelder P, Horvath S. WGCNA: An R package for weighted correlation network analysis. BMC Bioinformatics. 2008;9(1):559. 19114008 10.1186/1471-2105-9-559 PMC2631488

[37] Wu T, Hu E, Xu S, Chen M, Guo P, Dai Z, Feng T, Zhou L, Tang W, Zhan LI, et al. clusterProfiler 4.0: A universal enrichment tool for interpreting omics data. Int J Hydrogen Energ. 2021;2(3). 10.1016/j.xinn.2021.100141 PMC8454663 34557778

[38] Salihoglu R, Srivastava M, Liang C, Schilling K, Szalay A, Bencurova E, Dandekar T. PRO-Simat: Protein network simulation and design tool. Comput Struct Biotechnol J. 2023;21:2767–2779. 37181657 10.1016/j.csbj.2023.04.023 PMC10172639

[39] Avramopoulos D, Szymanski M, Wang R, Bassett S. Gene expression reveals overlap between normal aging and Alzheimer’s disease genes. Neurobiol Aging. 2011;32(12):2319-e27. 10.1016/j.neurobiolaging.2010.04.019 PMC2945399 20570407

[40] Peng S, Zeng L, Haure-Mirande JV, Wang M, Huffman DM, Haroutunian V, Ehrlich ME, Zhang B, Tu Z. Transcriptomic changes highly similar to Alzheimer’s disease are observed in a subpopulation of individuals during normal brain aging. Front Aging Neurosci. 2021;13: Article 711524. 34924992 10.3389/fnagi.2021.711524 PMC8675870

[41] Yuan Y, Chen YPP, Boyd-Kirkup J, Khaitovich P, Somel M. Accelerated aging-related transcriptome changes in the female prefrontal cortex. Aging Cell. 2012;11(5):894–901. 22783978 10.1111/j.1474-9726.2012.00859.x PMC3470704

[42] Green GS, Fujita M, Yang HS, Taga M, Cain A, McCabe C, Comandante-Lou N, White CC, Schmidtner AK, Zeng L, et al. Cellular communities reveal trajectories of brain ageing and Alzheimer’s disease. Nature. 2024;633(8030):634–645. 39198642 10.1038/s41586-024-07871-6 PMC11877878

[43] Mandrekar-Colucci S, Landreth GE. Nuclear receptors as therapeutic targets for Alzheimer’s disease. Expert Opin Ther Targets. 2011;15(9):1085–1097. 21718217 10.1517/14728222.2011.594043 PMC3156324

[44] Bonda DJ, Lee HP, Kudo W, Zhu X, Smith MA, Lee HG. Pathological implications of cell cycle re-entry in Alzheimer disease. Expert Rev Mol Med. 2010;12: Article e19. 20584423 10.1017/S146239941000150X PMC2922901

[45] Pandey N, Vinod PK. Model scenarios for cell cycle re-entry in Alzheimer’s disease. Iscience. 2022;25(7). 10.1016/j.isci.2022.104543 PMC9209725 35747391

[46] Zhang Y, Geng R, Liu M, Deng S, Ding J, Zhong H, Tu Q. Shared peripheral blood biomarkers for Alzheimer’s disease, major depressive disorder, and type 2 diabetes and cognitive risk factor analysis. Heliyon. 2023;9(3). 10.1016/j.heliyon.2023.e14653 PMC10040717 36994393

[47] Sanford SA, Miller LV, Vaysburd M, Keeling S, Tuck BJ, Clark J, Neumann M, Syanda V, James LC, WA ME. The type-I interferon response potentiates seeded tau aggregation and exacerbates tau pathology. Alzheimers Dement. 2024;20(2):1013–1025. 37849026 10.1002/alz.13493 PMC10916982

[48] Sanford SA, McEwan WA. Type-I interferons in Alzheimer’s disease and other tauopathies. Front Cell Neurosci. 2022;16: Article 949340. 35910253 10.3389/fncel.2022.949340 PMC9334774

[49] Bilkei-Gorzo A, Racz I, Michel K, Zimmer A. Diminished anxiety- and depression-related behaviors in mice with selective deletion of the Tac1 gene. J Neurosci. 2002;22(22):10046–10052. 12427862 10.1523/JNEUROSCI.22-22-10046.2002 PMC6757849

[50] He ZX, Yin YY, Xi K, Xing ZK, Cao JB, Liu TY, Liu L, He XX, Yu HL, Zhu XJ. Nucleus accumbens Tac1-expressing neurons mediate stress-induced anhedonia-like behavior in mice. Cell Rep. 2020;33(5). 10.1016/j.celrep.2020.108343 33147466

[51] Borbély É, Hajna Z, Simon G, Berger A, Paige C, Quinn J, Pintér E, Szolcsányi J, Helyes Z. Investigation of the role of preprotachykinin a and C (TAC1 and TAC_4_) gene-derived peptides in anxiety, stress and depression-like behaviour in mice. Front Neurosci. 2011.

[52] Zhu M, Tang M, Du Y. Identification of TAC1 associated with Alzheimer’s disease using a robust rank aggregation approach. J Alzheimer’s Dis. 2023;91(4):1339–1349. 36617784 10.3233/JAD-220950

[53] Swerdlow RH. Mitochondria and mitochondrial cascades in Alzheimer’s disease. J Alzheimer’s Dis. 2018;62(3):1403–1416. 29036828 10.3233/JAD-170585 PMC5869994

[54] Tasic B, Yao Z, Graybuck LT, Smith KA, Nguyen TN, Bertagnolli D, Goldy J, Garren E, Economo MN, Viswanathan S, et al. Shared and distinct transcriptomic cell types across neocortical areas. Nature. 2018;563(7729):72–78. 30382198 10.1038/s41586-018-0654-5 PMC6456269

[55] McClintick JN, McBride WJ, Bell RL, Ding ZM, Liu Y, Xuei X, Edenberg HJ. Gene expression changes in glutamate and GABA-A receptors, neuropeptides, ion channels, and cholesterol synthesis in the periaqueductal gray following binge-like alcohol drinking by adolescent alcohol-preferring (P) rats. Alcohol Clin Exp Res. 2016;40(5):955–968. 27061086 10.1111/acer.13056 PMC4844794

[56] Schank JR, Tapocik JD, Barbier E, Damadzic R, Eskay RL, Sun H, Rowe KE, King CE, Yao M, Flanigan ME, et al. Tacr1 gene variation and neurokinin 1 receptor expression is associated with antagonist efficacy in genetically selected alcohol-preferring rats. Biol Psychiatry. 2013;73(8):774–781. 23419547 10.1016/j.biopsych.2012.12.027 PMC3773538

[57] Nelson BS, Sequeira MK, Schank JR. Bidirectional relationship between alcohol intake and sensitivity to social defeat: Association with Tacr1 and Avp expression. Addict Biol. 2018;23(1):142–153. 28150369 10.1111/adb.12494 PMC5538906

[58] Dubic MG, Edwards S, McDaniel LS, Simon L, Molina PE. Differential regulation of tachykinin and opioid system gene expression in brain and immune cells of chronic binge alcohol-treated simian immunodeficiency virus-infected macaques. AIDS Res Hum Retrovir. 2023;39(5):232–240. 36762939 10.1089/aid.2022.0122 PMC10171953

[59] Terzenidou ME, Segklia A, Kano T, Papastefanaki F, Karakostas A, Charalambous M, Ioakeimidis F, Papadaki M, Kloukina I, Chrysanthou-Piterou M, et al. Novel insights into SLC25A46-related pathologies in a genetic mouse model. PLOS Genet. 2017;13(4): Article e1006656. 28376086 10.1371/journal.pgen.1006656 PMC5380310

[60] Nikolova YS, Iruku SP, Lin CW, Conley ED, Puralewski R, French B, Hariri AR, Sibille E. FRAS1-related extracellular matrix 3 (FREM3) single-nucleotide polymorphism effects on gene expression, amygdala reactivity and perceptual processing speed: An accelerated aging pathway of depression risk. Front Psychol. 2015;6:1377. 26441752 10.3389/fpsyg.2015.01377 PMC4584966

[61] Cai X, Li W, Shi W, Cai Y, Zhou J. Role of ferroptosis-related IREB2 in the shared genetic etiology between smoking and facial aging: Insights from large-scale genome-wide cross-trait analysis. Comput Struct Biotechnol J. 2025;27:3433–3442. 40799911 10.1016/j.csbj.2025.07.049 PMC12341527

[62] Xiong J, Xu T, Wang Z, Huang Y, Zhang S, Yang G, Yang J, Gao S, Wang T, Jian X, et al. The hidden genetic and microbial networks connecting neuropsychiatric and digestive disorders. Comput Struct Biotechnol J. 2025;27:3114–3126. 40703095 10.1016/j.csbj.2025.07.029 PMC12284710

[63] Zhang X, Song X, Hu G, Yang Y, Liu R, Zhou N, Basu S, Qiao D, Hou Q. Landscape of intrinsically disordered proteins in mental disorder diseases. Comput Struct Biotechnol J. 2024;23:3839–3849. 39534590 10.1016/j.csbj.2024.10.043 PMC11554586

[64] Swerdlow RH. The Alzheimer’s disease mitochondrial cascade hypothesis: A current overview. J Alzheimer’s Dis. 2023;92(3):751–768. 36806512 10.3233/JAD-221286

[65] Stevens B, Allen NJ, Vazquez LE, Howell GR, Christopherson KS, Nouri N, Micheva KD, Mehalow AK, Huberman AD, Stafford B, et al. The classical complement cascade mediates CNS synapse elimination. Cell. 2007;131(6):1164–1178. 18083105 10.1016/j.cell.2007.10.036

[66] Dai Z, Hu T, Wei J, Wang X, Cai C, Gu Y, Hu Y, Wang W, Wu Q, Fang J. Network-based identification and mechanism exploration of active ingredients against Alzheimer’s disease via targeting endoplasmic reticulum stress from traditional chinese medicine. Comput Struct Biotechnol J. 2024;23:506–519. 38261917 10.1016/j.csbj.2023.12.017 PMC10796977

[67] Luo L, Yan T, Yang L, Zhao M. Aluminum chloride and D-galactose induced a zebrafish model of Alzheimer’s disease with cognitive deficits and aging. Comput Struct Biotechnol J. 2024;23:2230–2239. 38827230 10.1016/j.csbj.2024.05.036 PMC11140485

[68] Subramanian A, Tamayo P, Mootha VK, Mukherjee S, Ebert BL, Gillette MA, Paulovich A, Pomeroy SL, Golub TR, Lander ES, et al. Gene set enrichment analysis: A knowledge-based approach for interpreting genome-wide expression profiles. Proc Natl Acad Sci USA. 2005;102(43):15545–15550. 16199517 10.1073/pnas.0506580102 PMC1239896

[69] Sun S, Yadav S, Pingili M, Chang D, Wang J. Estimating the effect of tissue-and blood-derived cell reference matrices on deconvolving bulk transcriptomic datasets. Comput Struct Biotechnol J. 2025;27:3579–3588. 40821716 10.1016/j.csbj.2025.07.058 PMC12356330

[70] Verlaan T, Bouland G, Mahfouz A, Reinders M. scAGG: Sample-level embedding and classification of Alzheimer’s disease from single-nucleus data. Comput Struct Biotechnol J. 2025;27:3753–3761. 40977903 10.1016/j.csbj.2025.08.009 PMC12448040

[71] Sanfilippo C, Castrogiovanni P, Imbesi R, Vecchio M, Sortino M, Musumeci G, Vinciguerra M, Di Rosa M. Exploring SERPINA3 as a neuroinflammatory modulator in Alzheimer’s disease with sex and regional brain variations. Metab Brain Dis. 2025;40(1):83. 39754632 10.1007/s11011-024-01523-4

[72] Mostafavi S, Gaiteri C, Sullivan SE, White CC, Tasaki S, Xu J, Taga M, Klein HU, Patrick E, Komashko V, et al. A molecular network of the aging human brain provides insights into the pathology and cognitive decline of Alzheimer’s disease. Nat Neurosci. 2018;21(6):811–819. 29802388 10.1038/s41593-018-0154-9 PMC6599633

[73] Wan YW, Al-Ouran R, Mangleburg CG, Perumal TM, Lee TV, Allison K, Swarup V, Funk CC, Gaiteri C, Allen M, et al. Meta-analysis of the Alzheimer’s disease human brain transcriptome and functional dissection in mouse models. Cell Rep. 2020;32(2):107908. 32668255 10.1016/j.celrep.2020.107908 PMC7428328

[74] De Jager PL, Ma Y, McCabe C, Xu J, Vardarajan BN, Felsky D, Klein HU, White CC, Peters MA, Lodgson B, et al. A multi-omic atlas of the human frontal cortex for aging and Alzheimer’s disease research. Sci Data. 2018;5(1): Article 180142. 30084846 10.1038/sdata.2018.142 PMC6080491

[75] Mathys H, Davila-Velderrain J, Peng Z, Gao F, Mohammadi S, Young JZ, Menon M, He L, Abdurrob F, Jiang X, et al. Single-cell transcriptomic analysis of Alzheimer’s disease. Nature. 2019;570(7761):332–337. 31042697 10.1038/s41586-019-1195-2 PMC6865822

[76] Sun N, Victor MB, Park YP, Xiong X, Scannail AN, Leary N, Prosper S, Viswanathan S, Luna X, Boix CA, et al. Human microglial state dynamics in Alzheimer’s disease progression. Cell. 2023;186(20):4386–4403. 37774678 10.1016/j.cell.2023.08.037 PMC10644954

[77] D’Alessandro MCB, Kanaan S, Geller M, Praticò D, Daher JPL. Mitochondrial dysfunction in Alzheimer’s disease. Ageing Res Rev. 2025;107: Article 102713. 40023293 10.1016/j.arr.2025.102713

[78] Patel T, Carnwath TP, Wang X, Allen M, Lincoln SJ, Lewis-Tuffin LJ, Quicksall ZS, Lin S, Tutor‐New FQ, Ho CC, et al. Transcriptional landscape of human microglia implicates age, sex, and APOE-related immunometabolic pathway perturbations. Aging Cell. 2022;21(5): Article e13606. 35388616 10.1111/acel.13606 PMC9124307

[79] Fabian D, Flatt T. The evolution of aging. Nat Educ Knowl. 2011;3:1–10.

[80] Xiang Y, Tanwar V, Singh P, La Follette L, Narayan VP, Kapahi P. Early menarche and childbirth accelerate aging-related outcomes and age-related diseases: Evidence for antagonistic pleiotropy in humans. Elife. 2025;13:RP102447. 40792619 10.7554/eLife.102447 PMC12342826

[81] Everman ER, Morgan TJ. Antagonistic pleiotropy and mutation accumulation contribute to age-related decline in stress response. Evolution. 2018;72(2):303–317. 29214647 10.1111/evo.13408

[82] Bekkos CH, Sujan MAJ, Stunes AK, Tari AR, Aagård N, Brobakken CL, Brevig MS, Syversen U, Wang E, Mosti MP. Acute effects of a single bout of high-intensity strength and endurance exercise on cognitive biomarkers in young adults and elderly men: A within-subjects crossover study. J Transl Med. 2025;23(1):685. 40537774 10.1186/s12967-025-06685-y PMC12177964

[83] Naumova OY, Palejev D, Vlasova NV, Lee M, Rychkov SY, Babich ON, Vaccarino FM, Grigorenko EL. Age-related changes of gene expression in the neocortex: Preliminary data on RNA-Seq of the transcriptome in three functionally distinct cortical areas. Dev Psychopathol. 2012;24(4):1427–1442. 23062308 10.1017/S0954579412000818 PMC3539811

[84] Hasenöhrl R, Huston J, Schuurman T. Neuropeptide substance P improves water maze performance in aged rats. Psychopharmacology. 1990;101(1):23–26. 1693002 10.1007/BF02253712

[85] Yu Y, Zeng C, Shu S, Liu X, Li C. Similar effects of substance P on learning and memory function between hippocampus and striatal marginal division. Neural Regen Res. 2014;9(8):857–863. 25206901 10.4103/1673-5374.131603 PMC4146251

[86] Li J, Li L, Cai S, Song K, Hu S. Identification of novel risk genes for Alzheimer’s disease by integrating genetics from hippocampus. Sci Rep. 2024;14(1):27484. 39523385 10.1038/s41598-024-78181-0 PMC11551212

[87] Li Z, Peng Y, Hufnagel RB, Hu YC, Zhao C, Queme LF, Khuchua Z, Driver AM, Dong F, Lu QR, et al. Loss of SLC25A46 causes neurodegeneration by affecting mitochondrial dynamics and energy production in mice. Hum Mol Genet. 2017;26(19):3776–3791. 28934388 10.1093/hmg/ddx262 PMC6074941

[88] Wang YT, Li D, Ang TFA, Xia W, Au R, Farrer LA, Stein TD, Jun GR. Gene expression differences between symptomatic and asymptomatic individuals with neuropathologically confirmed alzheimer’s disease. Alzheimers Dement. 2023;19: Article e064213.

[89] Mitra S, Bp K, CR S, Saikumar NV, Philip P, Narayanan M. Alzheimer’s disease rewires gene coexpression networks coupling different brain regions. npj Syst Biol Appl. 2024;10(1):50. 38724582 10.1038/s41540-024-00376-y PMC11082197

[90] Giorgi C, Aan FJ, Glibetic N, Ramaccini D, Modesti L, Vitto VA, Montoya-Uribe V, Corpuz A, Missiroli S, Simões I, et al. Mitochondrial PTRH2 controls the deubiquitinase TRABID to regulate mt-ND5 stability and metabolism. PNAS Nexus. 2025;4(6): Article pgaf178. 40496187 10.1093/pnasnexus/pgaf178 PMC12150287

[91] Hu H, Matter ML, Issa-Jahns L, Jijiwa M, Kraemer N, Musante L, de la Vega M, Ninnemann O, Schindler D, Damatova N, et al. Mutations in PTRH2 cause novel infantile-onset multisystem disease with intellectual disability, microcephaly, progressive ataxia, and muscle weakness. Ann Clin Transl Neurol. 2014;1(12):1024–1035. 25574476 10.1002/acn3.149 PMC4284127

[92] Sharkia R, Shalev SA, Zalan A, Marom-David M, Watemberg N, Urquhart JE, Daly SB, Bhaskar SS, Williams SG, Newman WG, et al. Homozygous mutation in PTRH2 gene causes progressive sensorineural deafness and peripheral neuropathy. Am J Med Genet A. 2017;173(4):1051–1055. 28328138 10.1002/ajmg.a.38140

[93] Bertram N, Izawa T, Thoma F, Schwenkert S, Duvezin-Caubet S, Park SH, Wagener N, Devin A, Osman C, Neupert W, et al. Delayed protein translocation protects mitochondria against toxic CAT-tailed proteins. Mol Cell. 2025;85(21):4082–4092. 41118763 10.1016/j.molcel.2025.09.030

[94] Witzel S, Huss A, Nagel G, Rosenbohm A, Rothenbacher D, Peter RS, Bäzner H, Börtlein A, Dempewolf S, Schabet M, et al. Population-based evidence for the use of serum neurofilaments as individual diagnostic and prognostic biomarkers in amyotrophic lateral sclerosis. Ann Neurol. 2024;96(6):1040–1057. 39177232 10.1002/ana.27054

[95] Avey DR, Ng B, Vialle RA, Kearns NA, de Paiva Lopes K, Iatrou A, De Tissera S, Vyas H, Saunders DM, Flood DJ, et al. Uncovering plaque-glia niches in human Alzheimer’s disease brains using spatial transcriptomics. Mol Neurodegener Adv. 2025;1(1):2. 40740481 10.1186/s44477-025-00002-z PMC12310243

