# Supplementary material for: Integrated Multi-Tissue Transcriptomics Reveals Antagonistic Pleiotropy in Aging and Alzheimer’s Disease
Source: Comput Struct Biotechnol J. 2026 Jun 8;35(1):0134. doi: 10.34133/csbj.0134 (PMC13243799; doi:10.34133/csbj.0134)
Supplement: Supplementary 1 — Figs. S1 to S11 Tables S1 to S3 [file csbj.0134.f1.zip › Supplementary Materials.docx]

**Supplementary Figure legends**

Supplementary Figure 1. Top differentially expressed genes between child and young-adult fibroblast samples. Heatmap of the top 50 differentially expressed genes in the child-versus-young-adult comparison. Columns represent samples and rows represent genes; colors indicate centered expression values. The top annotation indicates developmental group.

Supplementary Figure 2. GO biological process enrichment for child-versus-young-adult fibroblast DEGs. Dot plot of enriched GO biological process terms. GeneRatio is shown on the x-axis, dot size indicates gene count, and color indicates adjusted P value. Enriched terms mainly involved mitochondrial respiration, oxidative phosphorylation, electron transport, ATP synthesis, and cellular respiration.

Supplementary Figure 3. KEGG enrichment for child-versus-young-adult fibroblast DEGs. Bar plot of enriched KEGG pathways. Bar length indicates gene count and color indicates adjusted P value. Enriched pathways were dominated by mitochondrial and oxidative-metabolism-related terms, including oxidative phosphorylation, thermogenesis, and neurodegeneration-associated pathway annotations.

Supplementary Figure 4. Hallmark pathway comparison between aging and Alzheimer’s disease consensus signatures. Scatter plot of Hallmark normalized enrichment scores (NES) for aging and AD consensus signatures. Dashed lines at NES = 0 define shared and opposite-direction pathway quadrants. Points are colored by quadrant classification.

Supplementary Figure 5. WGCNA parameter-sensitivity audit. WGCNA robustness was assessed across 27 parameter settings by varying soft-thresholding power, minimum module size, and merge-cut height. (A) Strict eigengene-level AP-module counts. (B) Primary module gene-content stability measured by maximum Jaccard overlap. (C) Stability of the top 200 AP-ranked genes relative to the primary analysis. (D) Gene–module AP consistency comparing gene-level AP classes with strict module-level AP labels.

Supplementary Figure 6. GO enrichment analysis of module ME03. Bubble plots showing enriched GO-BP, GO-CC, and GO-MF terms for ME03. GeneRatio is shown on the x-axis, bubble size indicates gene count, and color indicates adjusted P value. Enriched terms involved stimulus detection, sensory perception, immune-related processes, and receptor/channel activity.

Supplementary Figure 7. GO enrichment analysis of module ME04. Bubble plots showing enriched GO-BP, GO-CC, and GO-MF terms for ME04. GeneRatio is shown on the x-axis, bubble size indicates gene count, and color indicates adjusted P value. Enriched terms involved sensory signaling, extracellular/cell-surface components, cytokine activity, antigen binding, and immune-related molecular functions.

Supplementary Figure 8. KEGG enrichment analysis of modules ME03 and ME04. Bubble plots showing enriched KEGG pathways for ME03 and ME04. GeneRatio is shown on the x-axis, bubble size indicates gene count, and color indicates adjusted P value. Enriched pathways included sensory signaling, cytokine–receptor interaction, xenobiotic metabolism, neuroactive ligand–receptor interaction, and immune-related pathways.

Supplementary Figure 9. Inferred transcription factor activity in aging and Alzheimer’s disease. Scatter plot of inferred TF activity NES values for aging and AD consensus signatures. Dashed lines at NES = 0 define shared and opposite-direction TF-activity quadrants. Points are colored by quadrant classification, and selected TFs with strong concordant or discordant patterns are labeled.

Supplementary Figure 10. Cell-state-aware sensitivity analysis of AD-associated expression effects. (A) Marker-based cell-state enrichment scores across diagnosis and brain region. (B) Correlation structure among estimated cell-state scores. (C) Comparison of AD log₂ fold-changes before and after cell-state adjustment. (D) AD effect estimates for prioritized AP candidates before and after adjustment.

Supplementary Figure 11. External validation of prioritized AP candidates. Validation of AP candidates across GSE118553, GSE33000, and GSE5281. (A) Summary of testability, directional concordance, nominal support, and FDR-level support. (B) External AD log₂FC values relative to AP-expected direction. (C) Cohort-by-gene validation map for top AP candidates. (D) Cross-cohort meta-evidence versus directional reproducibility.
