# Supplementary material for: Integrated Multi-Tissue Transcriptomics Reveals Antagonistic Pleiotropy in Aging and Alzheimer’s Disease
Source: Comput Struct Biotechnol J. 2026 Jun 8;35(1):0134. doi: 10.34133/csbj.0134 (PMC13243799; doi:10.34133/csbj.0134)
Supplement: Supplementary 1 — Figs. S1 to S11 Tables S1 to S3 [file csbj.0134.f1.zip › Supplementary_Figure-10.pdf]

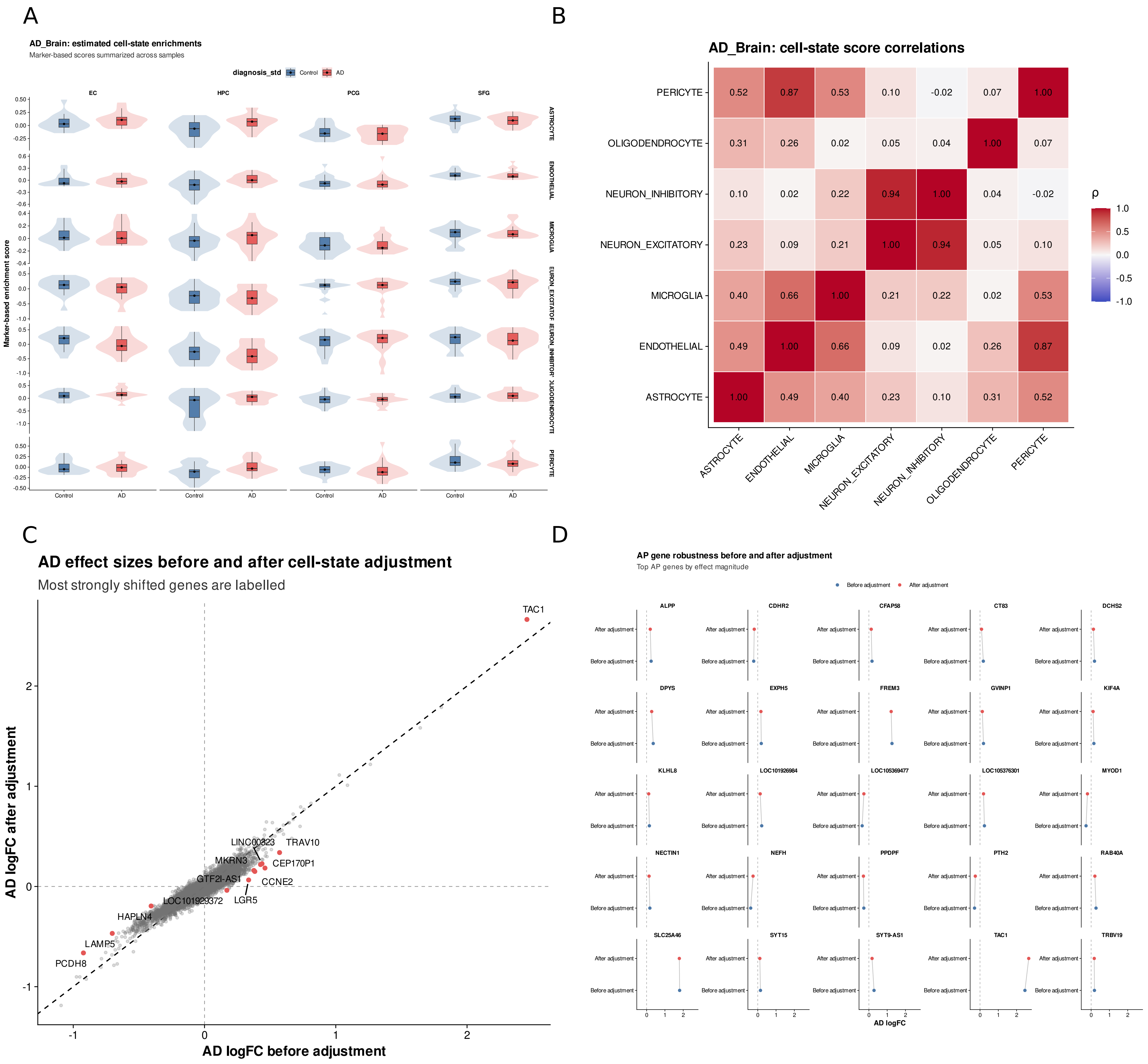
A
AD_Brain: estimated cell-state enrichments
Marker-based scores summarized across samples
diagnosis_std
Control
AD
EC
HPC
PCG
SFG
ASTROCYTE
ENDOTHELIAL
MICROGLIA
PERICYTE
Marker-based enrichment score
B
AD_Brain: cell-state score correlations
PERICYTE
OLIGODENDROCYTE
NEURON_INHIBITORY
NEURON_EXCITATORY
MICROGLIA
ENDOTHELIAL
ASTROCYTE
ρ
C
AD effect sizes before and after cell-state adjustment
Most strongly shifted genes are labelled
TAC1
LINC00823
TRAV10
MKRN3
CEP170P1
GTF2I-AS1
CCNE2
LOC101929372
LGR5
HAPLN4
LAMP5
PCDH8
AD logFC after adjustment
AD logFC before adjustment
D
AP gene robustness before and after adjustment
Top AP genes by effect magnitude
Before adjustment
After adjustment
ALPP
CDHR2
CFAP58
CT83
DCHS2
DPYS
EXPH5
FREM3
GVINP1
KIF4A
KLHL8
LOC101926984
LOC105369477
LOC105376301
MYOD1
NECTIN1
NEFH
PPDPF
PTH2
RAB40A
SLC25A46
SYT15
SYT9-AS1
TAC1
TRBV19
AD logFC
