# Supplementary material for: Integrated Multi-Tissue Transcriptomics Reveals Antagonistic Pleiotropy in Aging and Alzheimer’s Disease
Source: Comput Struct Biotechnol J. 2026 Jun 8;35(1):0134. doi: 10.34133/csbj.0134 (PMC13243799; doi:10.34133/csbj.0134)
Supplement: Supplementary 1 — Figs. S1 to S11 Tables S1 to S3 [file csbj.0134.f1.zip › Supplementary_Figure-11.pdf]

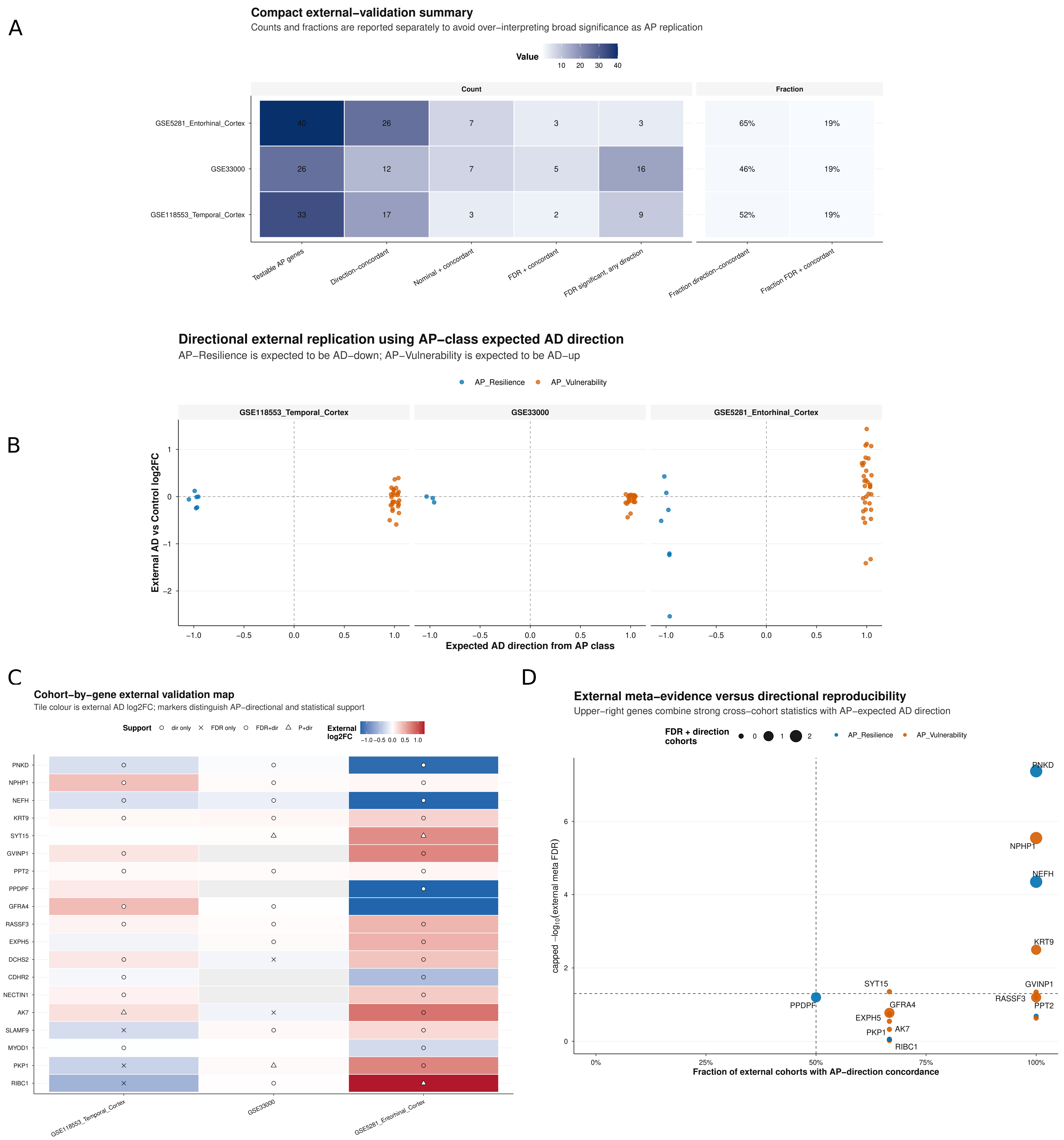

A
Compact external−validation summary
Counts and fractions are reported separately to avoid over−interpreting broad significance as AP replication
Value
10 20 30 40
Count
Fraction
GSE5281_Entorhinal_Cortex
GSE33000
GSE118553_Temporal_Cortex
40 26 7 3 3 65% 19%
26 12 7 5 16 46% 19%
33 17 3 2 9 52% 19%
Testable AP genes
Direction−concordant
Nominal + concordant
FDR + concordant
FDR significant, any direction
Fraction direction−concordant
Fraction FDR + concordant
B
Directional external replication using AP−class expected AD direction
AP−Resilience is expected to be AD−down; AP−Vulnerability is expected to be AD−up
AP_Resilience
AP_Vulnerability
GSE118553_Temporal_Cortex
GSE33000
GSE5281_Entorhinal_Cortex
External AD vs Control log2FC
Expected AD direction from AP class
C
Cohort−by−gene external validation map
Tile colour is external AD log2FC; markers distinguish AP−directional and statistical support
Support
dir only
FDR only
FDR+dir
P+dir
External log2FC
−1.0 −0.5 0.0 0.5 1.0
PNKD
NPHP1
NEFH
KRT9
SYT15
GVINP1
PPT2
PPDPF
GFRA4
RASSF3
EXPH5
DCHS2
CDHR2
NECTIN1
AK7
SLAMF9
MYOD1
PKP1
RIBC1
GSE118553_Temporal_Cortex
GSE33000
GSE5281_Entorhinal_Cortex
D
External meta−evidence versus directional reproducibility
Upper−right genes combine strong cross−cohort statistics with AP−expected AD direction
FDR + direction cohorts
0 1 2
AP_Resilience
AP_Vulnerability
capped −log10(external meta FDR)
Fraction of external cohorts with AP−direction concordance
0% 25% 50% 75% 100%
PNKD
NPHP1
NEFH
KRT9
SYT15
GVINP1
RASSF3
PPT2
PPDPF
GFRA4
EXPH5
AK7
PKP1
RIBC1
