# Supplementary material for: Integrated Multi-Tissue Transcriptomics Reveals Antagonistic Pleiotropy in Aging and Alzheimer’s Disease
Source: Comput Struct Biotechnol J. 2026 Jun 8;35(1):0134. doi: 10.34133/csbj.0134 (PMC13243799; doi:10.34133/csbj.0134)
Supplement: Supplementary 1 — Figs. S1 to S11 Tables S1 to S3 [file csbj.0134.f1.zip › Supplementary_Figure-5.pdf]

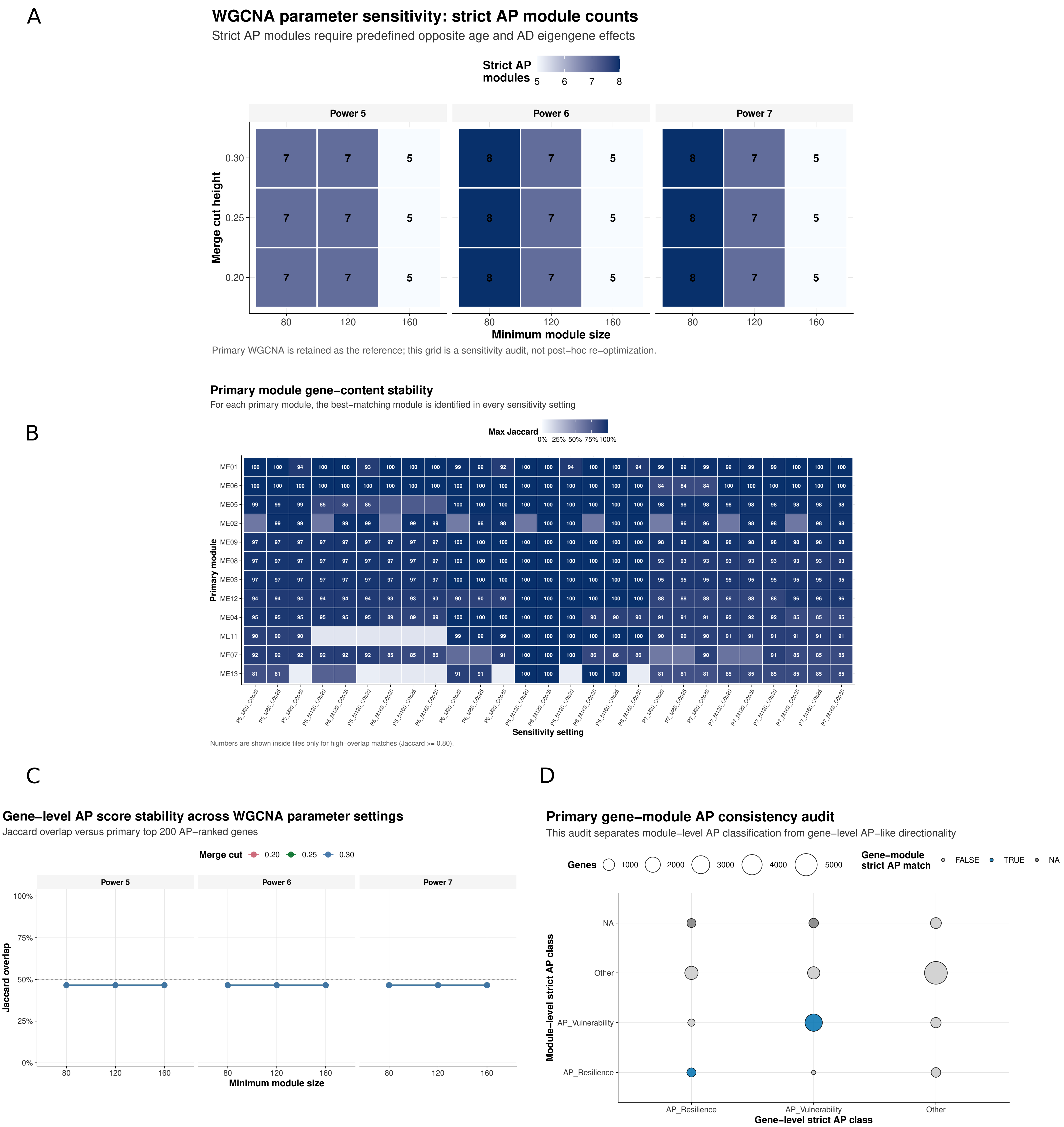
A
WGCNA parameter sensitivity: strict AP module counts
Strict AP modules require predefined opposite age and AD eigengene effects
Strict AP modules
5 6 7 8
Power 5
Power 6
Power 7
Merge cut height
0.30
0.25
0.20
80 120 160
Minimum module size
Primary WGCNA is retained as the reference; this grid is a sensitivity audit, not post−hoc re−optimization.
B
Primary module gene−content stability
For each primary module, the best−matching module is identified in every sensitivity setting
Max Jaccard
0% 25% 50% 75% 100%
Primary module
ME01 ME06 ME05 ME02 ME09 ME08 ME03 ME12 ME04 ME11 ME07 ME13
Sensitivity setting
Numbers are shown inside tiles only for high−overlap matches (Jaccard >= 0.80).
C
Gene−level AP score stability across WGCNA parameter settings
Jaccard overlap versus primary top 200 AP−ranked genes
Merge cut 0.20 0.25 0.30
Power 5
Power 6
Power 7
Jaccard overlap
100% 75% 50% 25% 0%
80 120 160
Minimum module size
D
Primary gene−module AP consistency audit
This audit separates module−level AP classification from gene−level AP−like directionality
Genes 1000 2000 3000 4000 5000
Gene−module strict AP match FALSE TRUE NA
Module−level strict AP class
NA
Other
AP_Vulnerability
AP_Resilience
AP_Resilience AP_Vulnerability Other
Gene−level strict AP class
