# Supplementary figures and images for: Integrated Multi-Tissue Transcriptomics Reveals Antagonistic Pleiotropy in Aging and Alzheimer’s Disease
Source: Comput Struct Biotechnol J. 2026 Jun 8;35(1):0134. doi: 10.34133/csbj.0134 (PMC13243799; doi:10.34133/csbj.0134)

Top 50 DE genes (Child vs Adult)

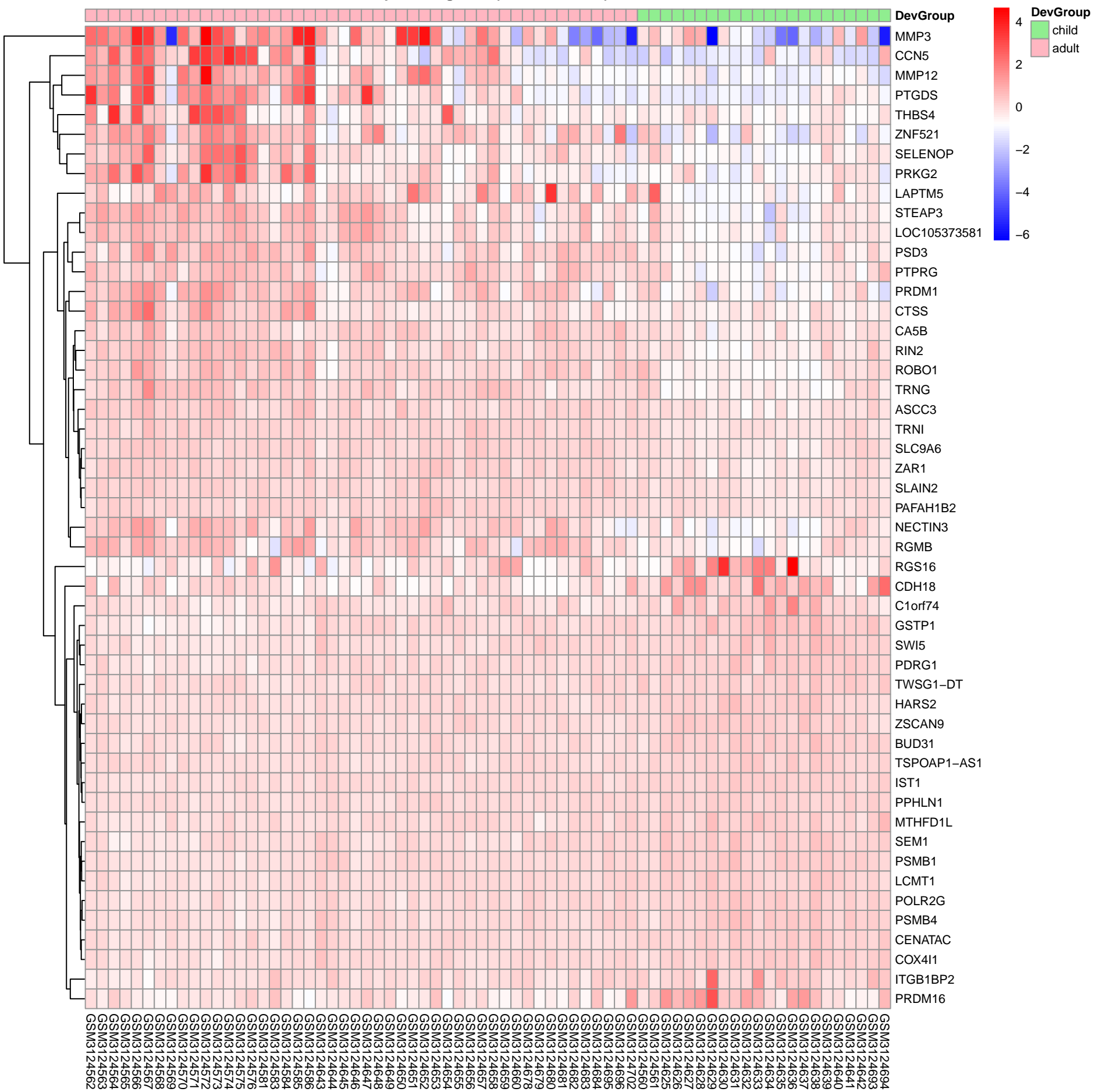

Supplement: Supplementary 1 — Figs. S1 to S11 Tables S1 to S3 [file csbj.0134.f1.zip › Supplementary_Figure-1.pdf]

# GO BP Enrichment (Child vs Adult)

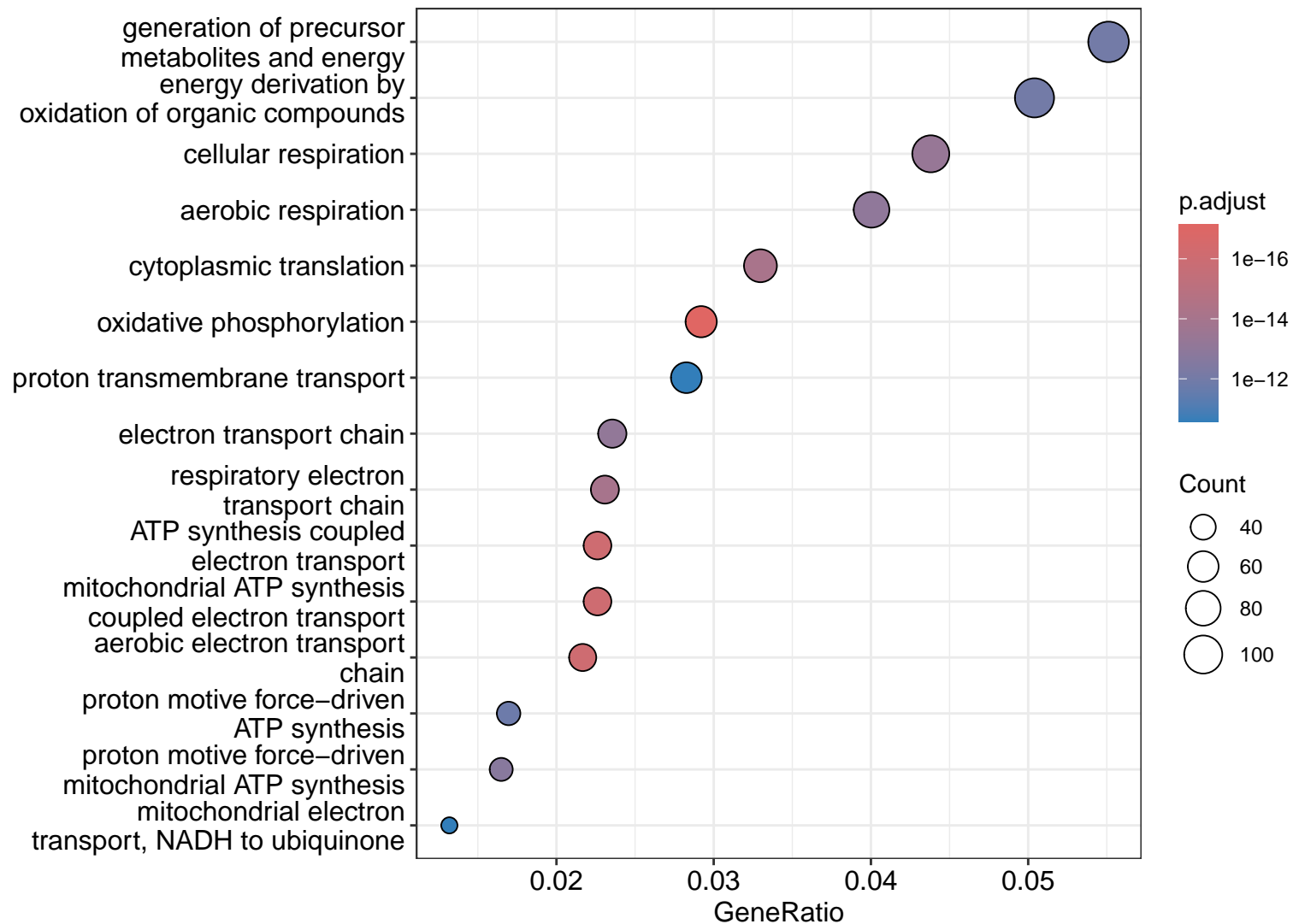

Supplement: Supplementary 1 — Figs. S1 to S11 Tables S1 to S3 [file csbj.0134.f1.zip › Supplementary_Figure-2.pdf]

## KEGG Enrichment (Child vs Adult)

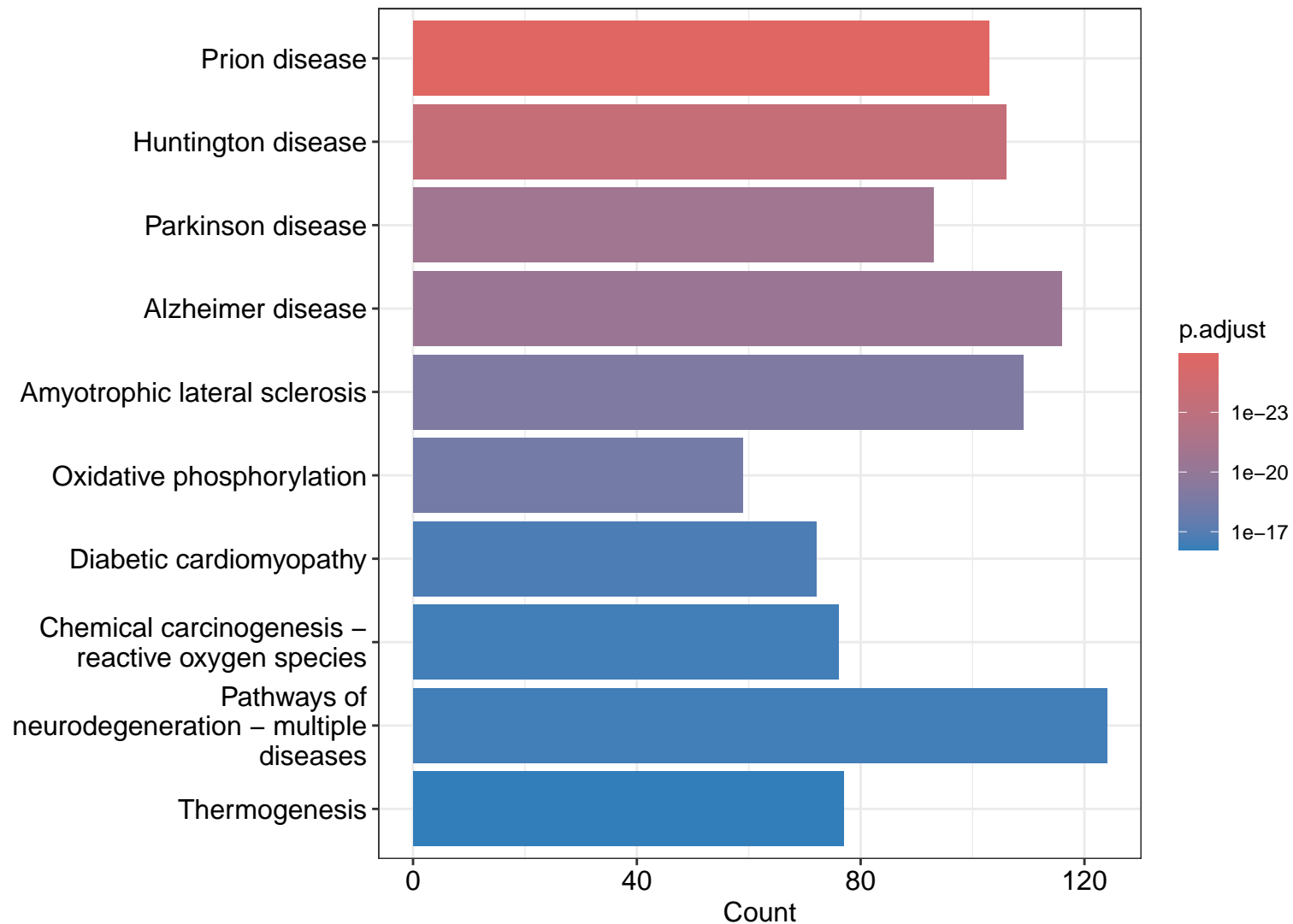

Supplement: Supplementary 1 — Figs. S1 to S11 Tables S1 to S3 [file csbj.0134.f1.zip › Supplementary_Figure-3.pdf]

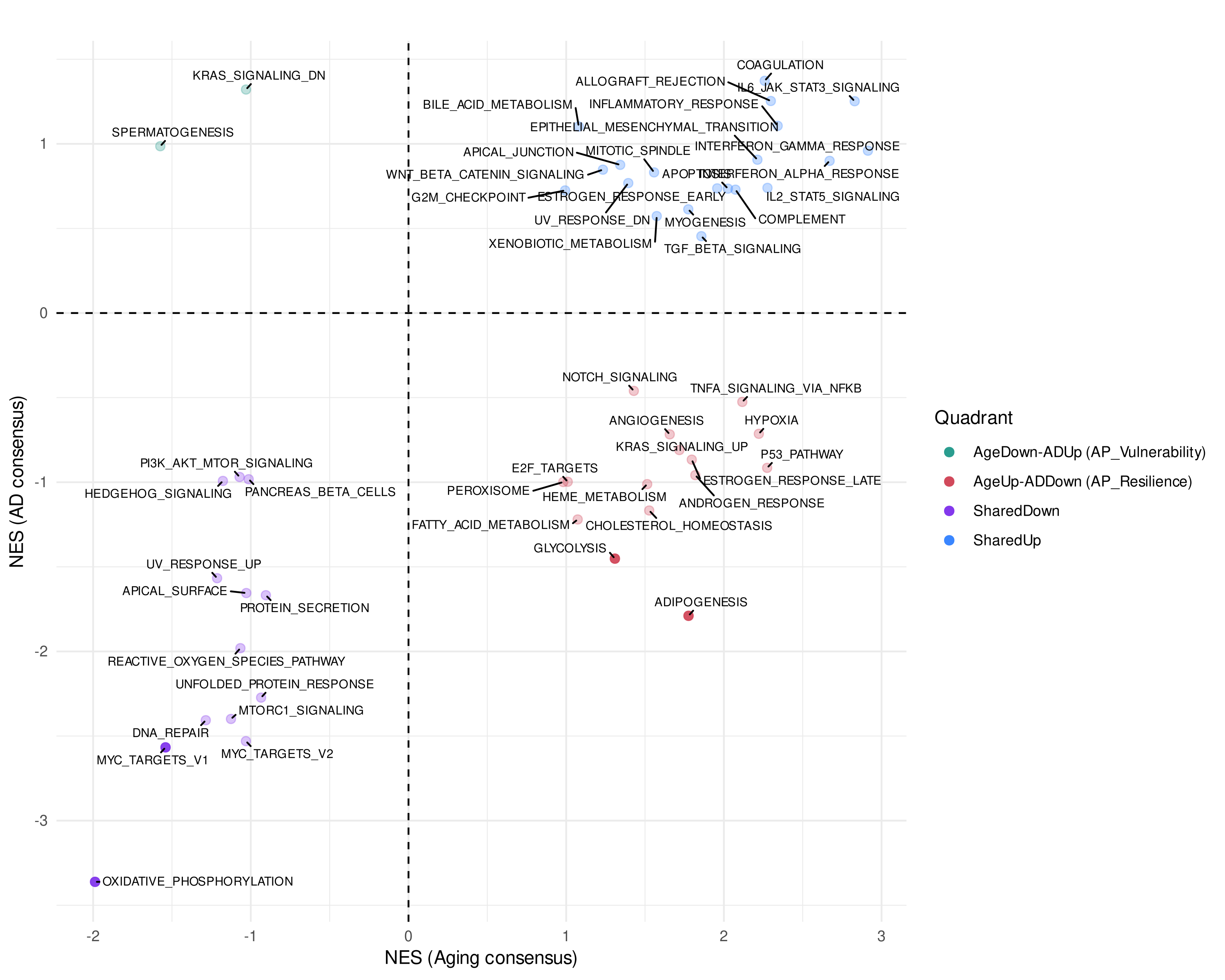

Supplement: Supplementary 1 — Figs. S1 to S11 Tables S1 to S3 [file csbj.0134.f1.zip › Supplementary_Figure-4.png]

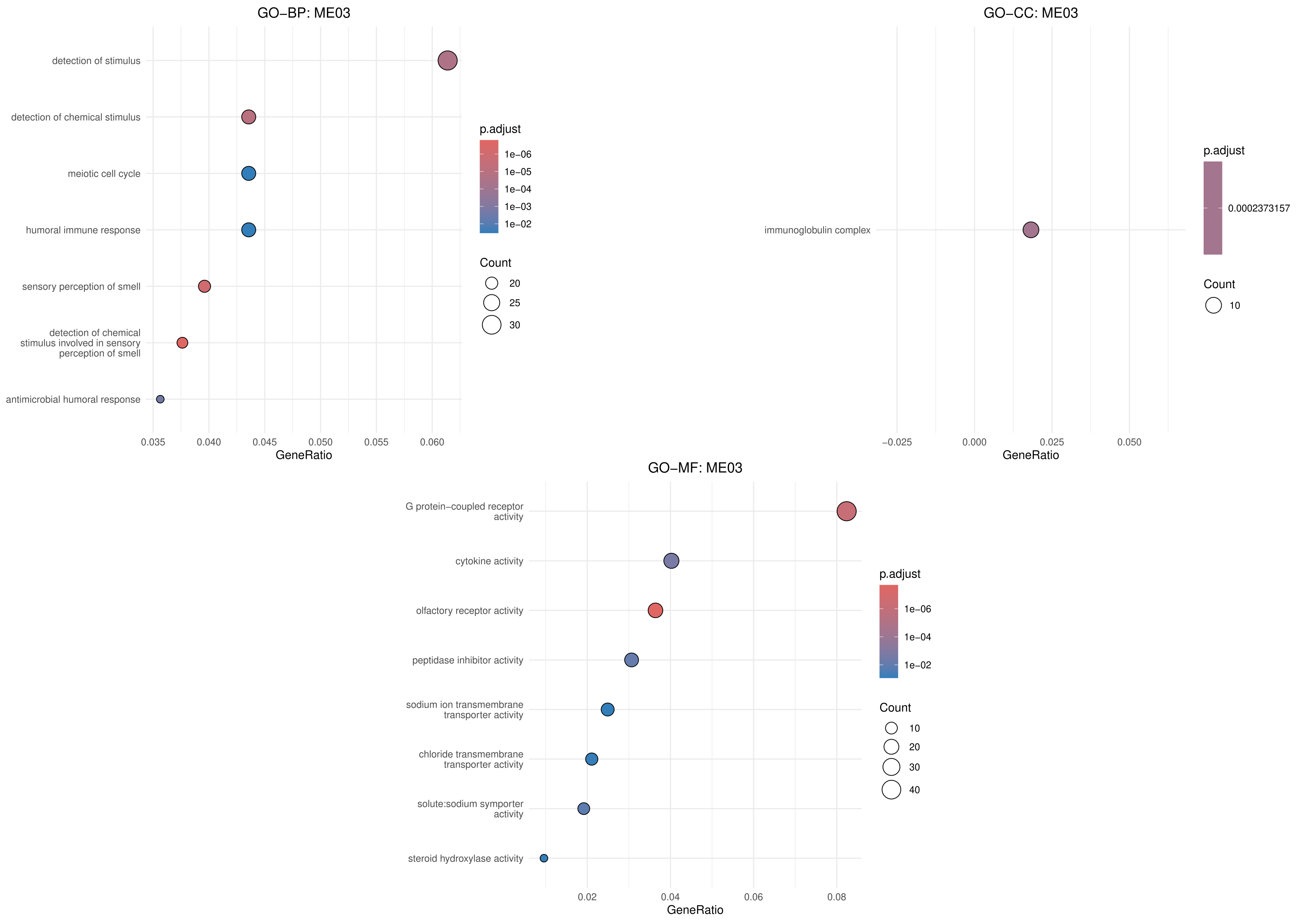

Supplement: Supplementary 1 — Figs. S1 to S11 Tables S1 to S3 [file csbj.0134.f1.zip › Supplementary_Figure-6.png]

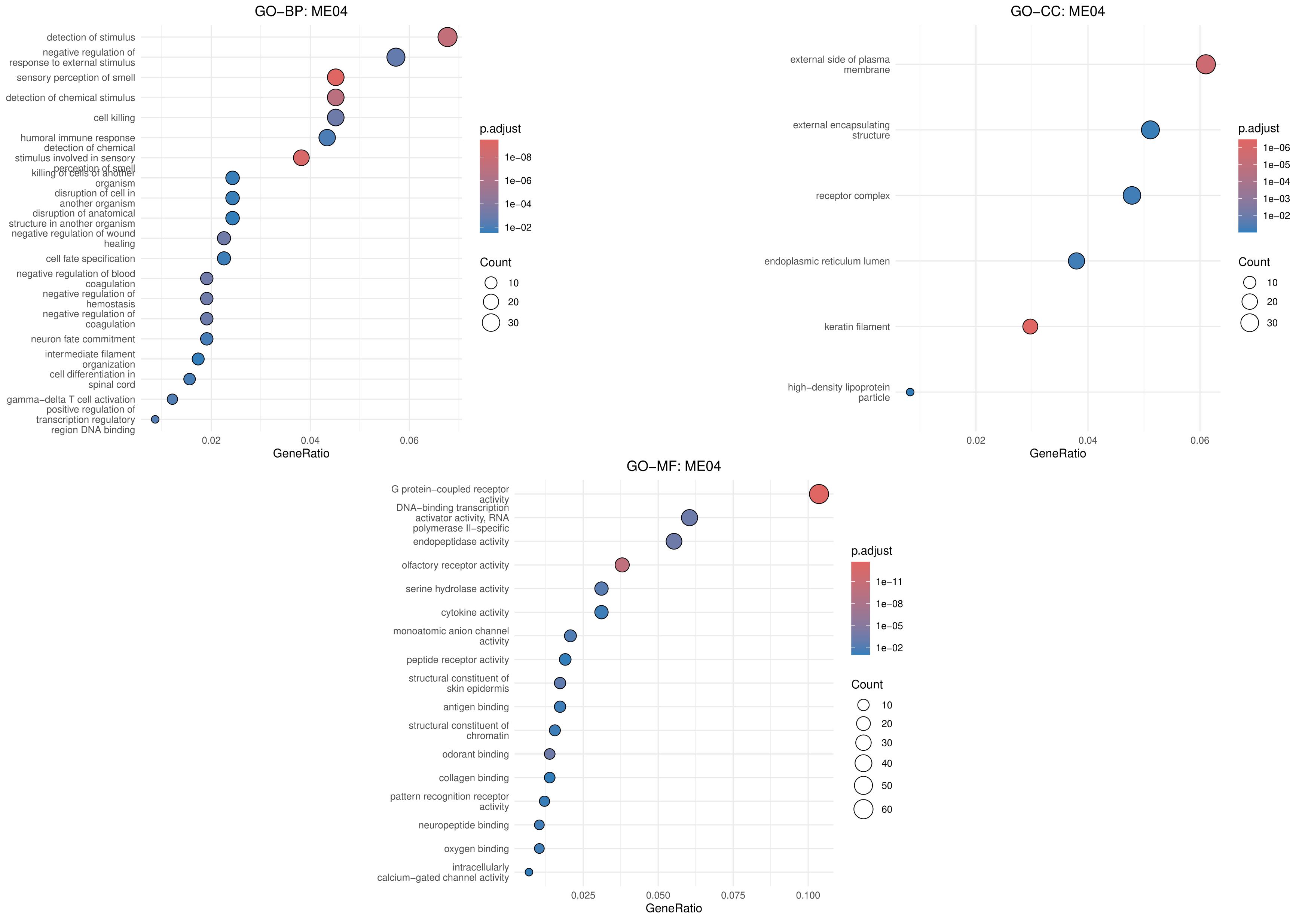

Supplement: Supplementary 1 — Figs. S1 to S11 Tables S1 to S3 [file csbj.0134.f1.zip › Supplementary_Figure-7.png]

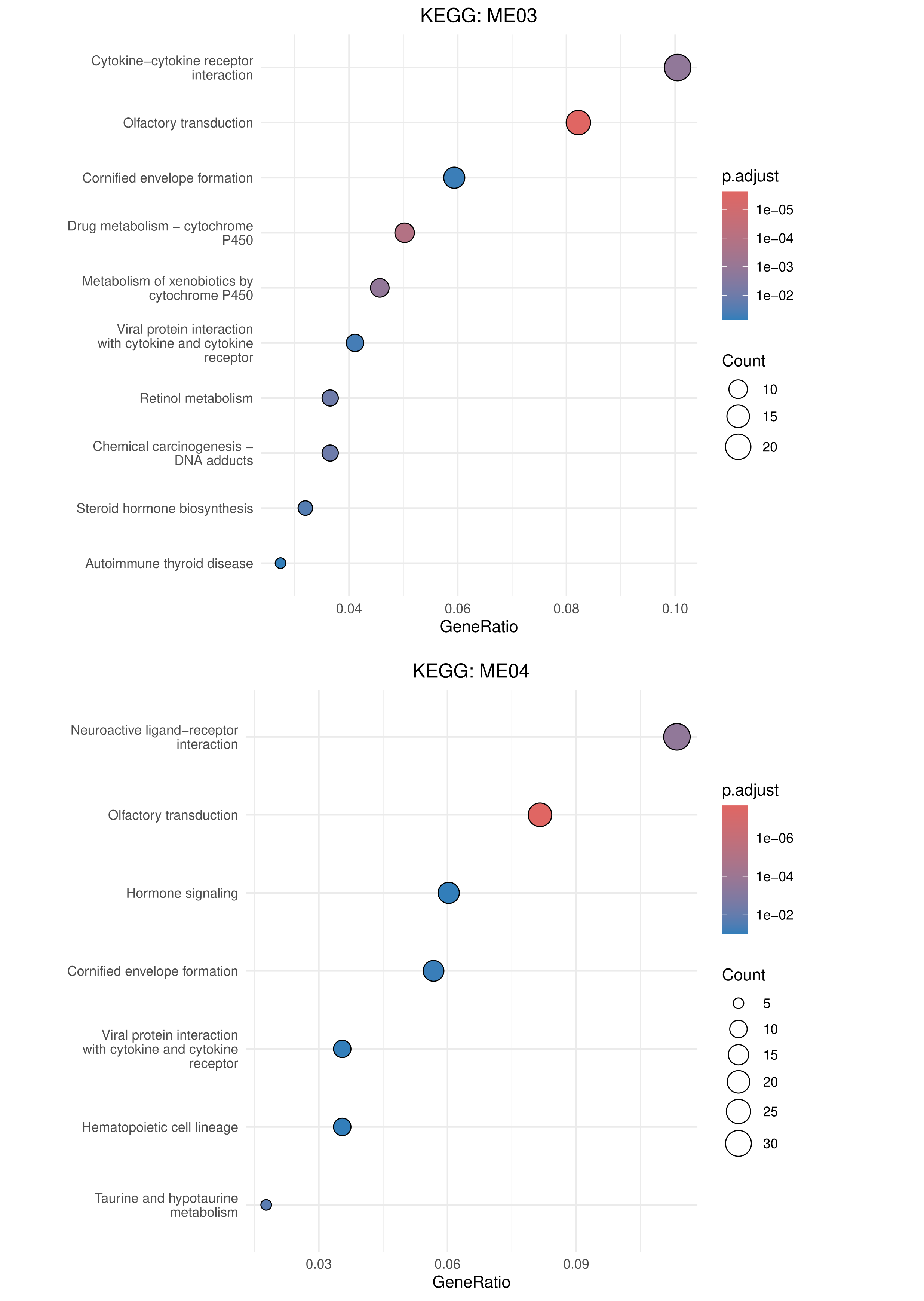

Supplement: Supplementary 1 — Figs. S1 to S11 Tables S1 to S3 [file csbj.0134.f1.zip › Supplementary_Figure-8.png]

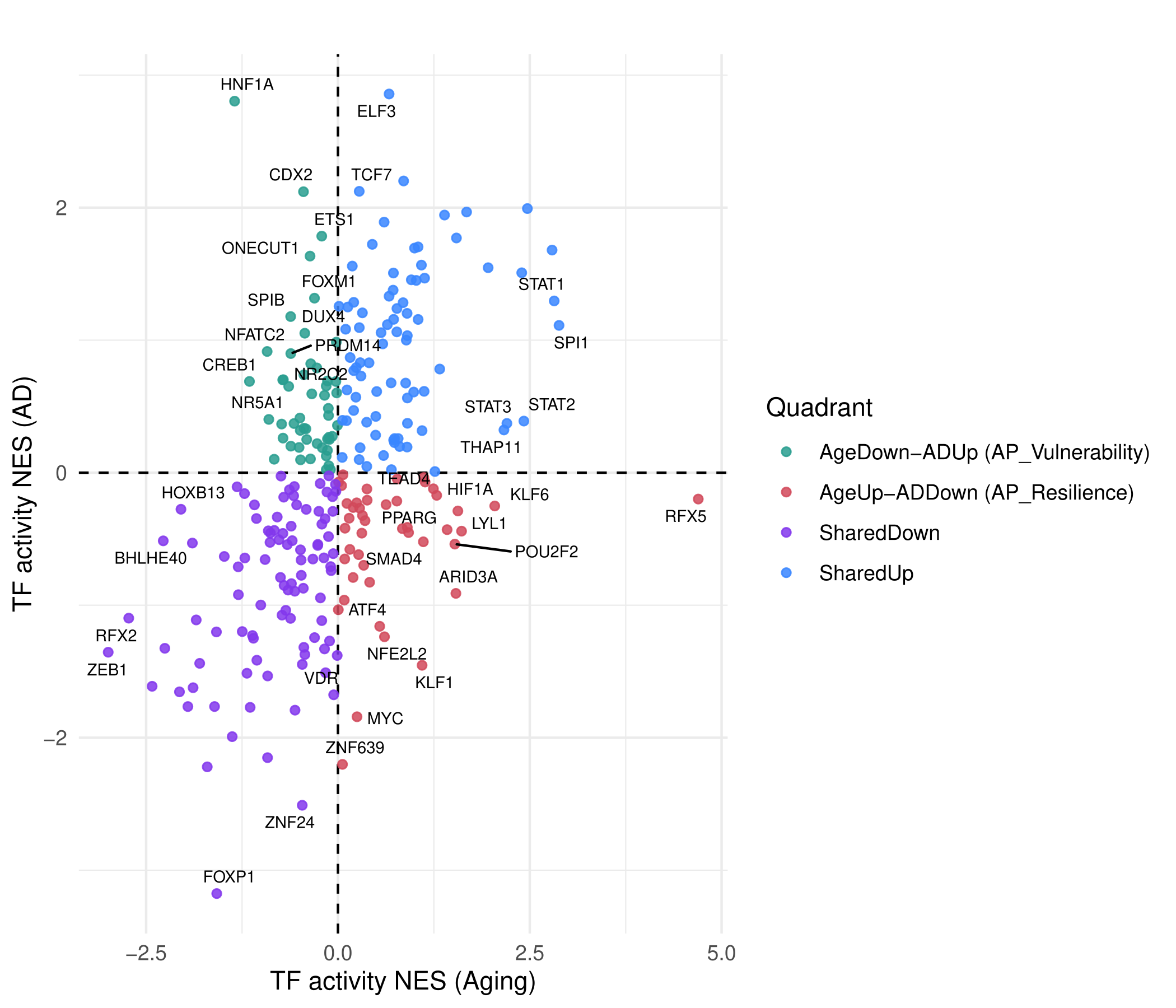

Supplement: Supplementary 1 — Figs. S1 to S11 Tables S1 to S3 [file csbj.0134.f1.zip › Supplementary_Figure-9.png]
